# Structural basis of nucleosome deacetylation and DNA linker tightening by Rpd3S histone deacetylase complex

**DOI:** 10.1038/s41422-023-00869-1

**Published:** 2023-09-04

**Authors:** Shuqi Dong, Huadong Li, Meilin Wang, Nadia Rasheed, Binqian Zou, Xijie Gao, Jiali Guan, Weijie Li, Jiale Zhang, Chi Wang, Ningkun Zhou, Xue Shi, Mei Li, Min Zhou, Junfeng Huang, He Li, Ying Zhang, Koon Ho Wong, Xiaofei Zhang, William Chong Hang Chao, Jun He

**Affiliations:** 1 CAS Key Laboratory of Regenerative Biology, Guangdong Provincial Key Laboratory of Stem Cell and Regenerative Medicine, GIBH-HKU Guangdong–Hong Kong Stem Cell and Regenerative Medicine Research Centre, GIBH-CUHK Joint Research Laboratory on Stem Cell and Regenerative Medicine, Guangzhou Institutes of Biomedicine and Health, Chinese Academy of Sciences, Guangzhou, Guangdong China; 2 University of Chinese Academy of Sciences, Beijing, China; 3 Faculty of Health Sciences, University of Macau, Macau SAR, China; 4 Key Laboratory of Biological Targeting Diagnosis, Therapy and Rehabilitation of Guangdong Higher Education Institutes, The Fifth Affiliated Hospital of Guangzhou Medical University, Guangzhou, Guangdong China; 5 Tomas Lindahl Nobel Laureate Laboratory, The Seventh Affiliated Hospital, Sun Yat-Sen University, Shenzhen, Guangdong China; 6 School of Life Sciences, University of Science and Technology of China, Hefei, Anhui China; 7 Guangzhou Laboratory, Guangzhou International Bio Island, Guangzhou, Guangdong China

**Keywords:** Cryoelectron microscopy, Nucleosomes, Histone post-translational modifications

## Abstract

In *Saccharomyces cerevisiae*, cryptic transcription at the coding region is prevented by the activity of Sin3 histone deacetylase (HDAC) complex Rpd3S, which is carried by the transcribing RNA polymerase II (RNAPII) to deacetylate and stabilize chromatin. Despite its fundamental importance, the mechanisms by which Rpd3S deacetylates nucleosomes and regulates chromatin dynamics remain elusive. Here, we determined several cryo-EM structures of Rpd3S in complex with nucleosome core particles (NCPs), including the H3/H4 deacetylation states, the alternative deacetylation state, the linker tightening state, and a state in which Rpd3S co-exists with the Hho1 linker histone on NCP. These structures suggest that Rpd3S utilizes a conserved Sin3 basic surface to navigate through the nucleosomal DNA, guided by its interactions with H3K36 methylation and the extra-nucleosomal DNA linkers, to target acetylated H3K9 and sample other histone tails. Furthermore, our structures illustrate that Rpd3S reconfigures the DNA linkers and acts in concert with Hho1 to engage the NCP, potentially unraveling how Rpd3S and Hho1 work in tandem for gene silencing.

## Introduction

The longevity of eukaryotes is negatively impacted by cryptic transcription, which is suppressed by the action of the Rpd3S/Sin3B corepressor complex.^1–4^ Reduced potassium dependency-3 small complex (Rpd3S), a conserved Sin3 histone deacetylase (HDAC) complex in *Saccharomyces cerevisiae*, is recruited to genes’ coding regions by phosphorylated the C-terminal domain (CTD) of RNA polymerase II (RNAPII) and stimulated by Set2-modified H3K36me3 to deacetylate chromatin.^1–3,5–8^ The coding regions are highly marked with H3K36me3, which is specifically recognized by the combined action of the Eaf3 and Rco1 subunits of Rpd3S.^2,9^ In contrast, the promoter regions are commonly labeled with H3K4me3, which is recognized by the Cti6 and Pho23 subunits in the Rpd3 large complex (Rpd3L).^10,11^ The differential histone methylation marks allow the Rpd3S and Rpd3L to utilize different subunits to target specific genomic locations for transcription regulation.

The five-subunit Rpd3S complex contains a unique subunit Rco1, three core subunits (Rpd3, Sin3, and Ume1) that are shared between the 12-subunit Rpd3L complex, and an Eaf3 subunit that is also a component of the NuA4 histone acetyltransferase (HAT).^1,12–16^ Rpd3 is a class-I histone deacetylase, which binds to the large Sin3 base in Rpd3S^1,2,5,13,14,17^ and targets all 4 histone tails.^18–21^ Apart from its deacetylase activity, Rpd3S also functions as an HDAC-independent chromatin stabilizer and prevents nucleosome eviction by chromatin remodeler RSC.^8^ The chromatin regulator function of Rpd3S is consistent with the observation that H3K36me3 suppresses histone exchange over coding regions to suppress cryptic transcription^22^ and that Rpd3S opposes the functions of both FACT and the Spt6–Spn1 transcription elongation complex in vivo.^15,23^

In eukaryotes, gene silencing is maintained by the engagement of H1 linker histone to facilitate the formation of higher-order nucleosome arrays post-transcription.^24–26^ Ume6, a component of Rpd3L, promotes Hho1 (H1 homologue in *Saccharomyces cerevisiae*) binding to the meiotic gene promoters.^27^ Furthermore, an *hho1Δrpd3Δ* double mutant results in additive derepression of early meiotic gene transcription, thus implying that Rpd3 works together with Hho1 to stabilize the repressive chromatin structure established by Rpd3L and Rpd3S.^8,27^ It is therefore conceivable that there is a transition from Rpd3S-mediated cryptic transcription repression to Hho1-mediated chromatin compaction.^28^

The recent studies on Rpd3S and Rpd3L revealed the complex assemblies and architecture in their apo states.^29–31^ Several structural studies have focused on the HDACs’ recognition of nucleosome substrates.^32,33^ The structure of Sirt6 was believed to be the first HDAC in a complex with an H3 tail substrate in the context of nucleosome.^32^ However, Sirt6 prefers H3 acetylated nucleosome over other histone acetylation, which significantly contrasts with other HDAC complexes (CoREST, MIDAC, Sin3, NuRD).^29,30,32^

Despite its fundamental role in epigenetic regulation, the mechanism by which Rpd3S deacetylates histone tails and how it coordinates with other factors to control the chromatin dynamics during transcription is still poorly understood. To gain mechanistic insights, we reconstituted the five-subunit Rpd3S complex in vitro and obtained a 3.5 Å structure using cryo-electron microscopy (cryo-EM). To capture different dynamic states of Rpd3S on NCP (nucleosome core particle), we further applied the following modifications in different combinations on the NCP: i) the addition of DNA linker(s) to NCP; ii) a methylated-lysine analog (MLA) at H3K36 to mimic trimethylation; and iii) a lysine-to-glutamine mutation at H3K9 (K9Q) to mimic an acetyl-lysine substrate. The modified NCPs were combined with Rpd3S to create four complexes namely Rpd3S–NCP^187bp/MLA^, Rpd3S–NCP^187bp/MLA/K9Q^, Rpd3S–NCP^167bp/MLA^, and Rpd3S–NCP^187bp^. Our structures reveal how Rpd3S potentially navigate through the core and extra nucleosomal DNA on the NCP, guided by its interaction with the acidic patch as well as H3K36MLA to sample and target the histone tails for deacetylation. During the process of deacetylation, Rpd3S further tightens the nucleosomal DNA linkers, which probably precludes the binding of transcription factors at cryptic transcription start sites. To further explore the transition from Rpd3S-mediated cryptic transcription repression to Hho1-mediated chromatin compaction, we determined an Rpd3S–NCP^187bp^–Hho1 complex structure with clear Hho1 density at the canonical dyad binding site of H1. The combined results lead to a model that Rpd3S not only deacetylates the NCP, but also acts in concert with Hho1 to engage the NCP and reconfigure the extra-nucleosomal DNA linkers, thereby promoting gene silencing.

## Results

### Architecture of Rpd3S

**Fig. 1:**
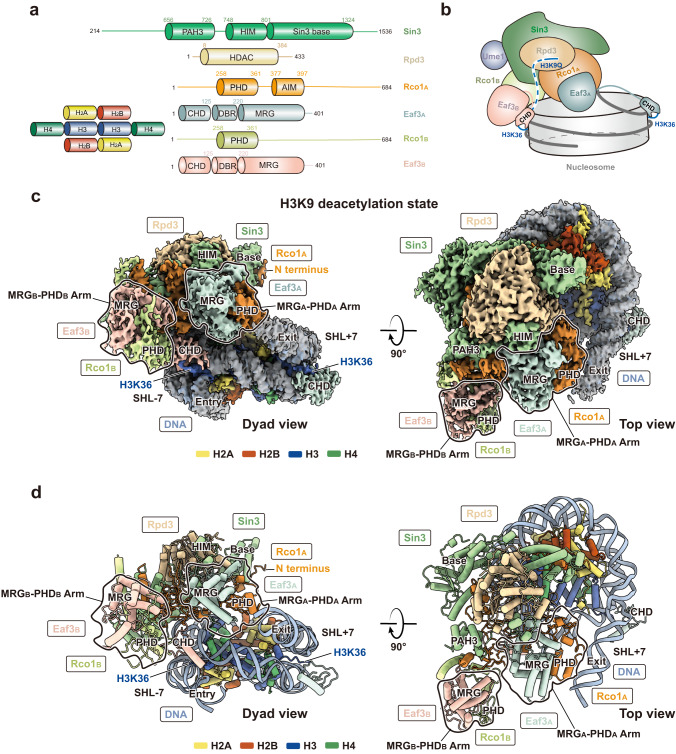
Overall structure of the Rpd3S complex bound to the nucleosome in the H3K9 deacetylation state. **a** Domain organization of Rpd3S subunits and histones. **b** Model of assembly and nucleosome recognition of Rpd3S complex in the H3K9 deacetylation state. Two Rco1 subunits and two Eaf3 subunits are indicated. Ume1 is schematically illustrated to connect to Sin3. Two CHDs of Eaf3_A/B_ are illustrated to interact with H3K36me3 tails. **c** Dyad view (left) and top view (right) of the cryo-EM composite map of the Rpd3S–nucleosome complex in its H3K9 deacetylation state. Two MRG-PHD arms are illustrated with bold contours. **d** Corresponding views of the cylindrical models are shown. In all maps and models, the subunits are colored as shown in **a**.

The five-subunit Rpd3S complex (Rpd3, Sin3 (residues 214–1536), Ume1, Rco1, and Eaf3) was reconstituted using a baculovirus/insect-cell expression system (Fig. 1a; Supplementary information, Fig. S1a).^34^ Multi-angle light scattering (MALS) yielded an experimental molecular weight of 528 kDa, which translates to a stoichiometry of a Sin3–Rpd3–Ume1–Rco1_A_–Eaf3_A_–Rco1_B_–Eaf3_B_ complex (Supplementary information, Fig. S2a). To visualize the structural architecture of Rpd3S, cryo-EM structure determination was performed using the purified Rpd3S complex to generate a three-dimensional (3D) map of 3.5 Å (Supplementary information, Figs. S1a, S3a, S9 and Table S1). The electron density of the Rpd3S structure allowed us to accurately define the Rpd3, Sin3, Rco1_A/B_, and Eaf3_A/B_ subunits. No density can accommodate the predicted WD40 domain structure of the Ume1 subunit in this high-resolution map. However, extended classification and refinement with a lowpass filter result in a Ume1 WD40 domain density (Supplementary information, Fig. S3b, c). The overall structure of Rpd3S reveals that the Sin3, Rco1_A_, and Eaf3_A_ subunits form a large continuous scaffold to hold the Rpd3 deacetylase in position (Fig. 1c). Rpd3 contacts an extended conserved motif of Sin3 (residues 748–801) that we term the histone-interacting motif (HIM) as it coordinates the H3 tail substrate binding (see later sections) (Supplementary information, Fig. S4a, b). HIM crisscrosses the Rpd3 deacetylase active site and connects to the Sin3 base (residues 801–1324), whose solution structure was partially determined in a complex with an Sds3 peptide by NMR previously.^35^ The equivalent binding site of the Sds3 peptide is occupied by the Rco1_A_ N terminus (residues 33–66) as revealed by de novo model building into the corresponding density and further validated by cross-linking mass spectrometry (XL-MS) (Supplementary information, Fig. S2b). Rco1_A_ N terminus binds to the Sin3 base to form an elongated scaffold, which makes extensive contact with Rpd3 and the rest of Rco1_A_ (Fig. 1c, d; Supplementary information, Fig. S5).

At the center of the Rpd3S structure is Rco1_A_ (residues 258–561), which contains the PHD-SID domain (plant homeobox domain and Sin3-interacting domain) (residues 260–374) and the conserved acidic patch-interacting motif (AIM) (residues 377–397) (Supplementary information, Figs. S5, S6a, b). Rco1_A_ coordinates Rpd3, Sin3, and the MRG domain of Eaf3_A_. Removal of Eaf3 from Rpd3S (Rpd3S^ΔEaf3^) results in the formation of a core complex of Rpd3–Sin3–Ume1 without Rco1 (Supplementary information, Fig. S1b). AIM coordinates the Sin3 HIM in the apo Rpd3S structure but changes its conformation to bind to the NCP’s acidic patch in the alternative deacetylation state (see later sections). The C terminus of Rco1_A_ consists of a long helix, which interacts with the equivalent helical segment of Rco1_B_. This helical interaction connects Rco1_B_–Eaf3_B_ to Rpd3S (Supplementary information, Fig. S1c). Both MRG domains of Eaf3 adopt identical structures and bind to the respective PHD-SID domains of Rco1_A_ and Rco1_B_, forming two MRG-PHD arms (Supplementary information, Figs. S1c, S6c). The density of the N-terminal chromodomains (CHDs) of the two Eaf3 is not visible, suggesting that their CHDs are likely mobile in the absence of the nucleosome substrate.

### Rpd3S engages NCP via multiple contacts at superhelical location (SHL) **+** 2 for H3/H4 deacetylation

Previous studies indicated that Rpd3S exhibits higher affinity towards NCP containing extra DNA linkers and the H3K36 methylation.^2,20,36^ To understand how these nucleosomal features impact Rpd3S binding to the NCP, we reconstituted an NCP with two extra 20 bp DNA linkers flanking the Widom 601 sequence (187 bp) and an MLA at the lysine-36 position of the H3 histone to mimic a tri-methylated lysine (H3K36MLA) (Supplementary information, Fig. S2c).^37^ The resulting NCP^187bp/MLA^ was used to form a complex with Rpd3S under gradient fixation (GraFix) conditions for cryo-EM structure determination.^38^

**Fig. 2:**
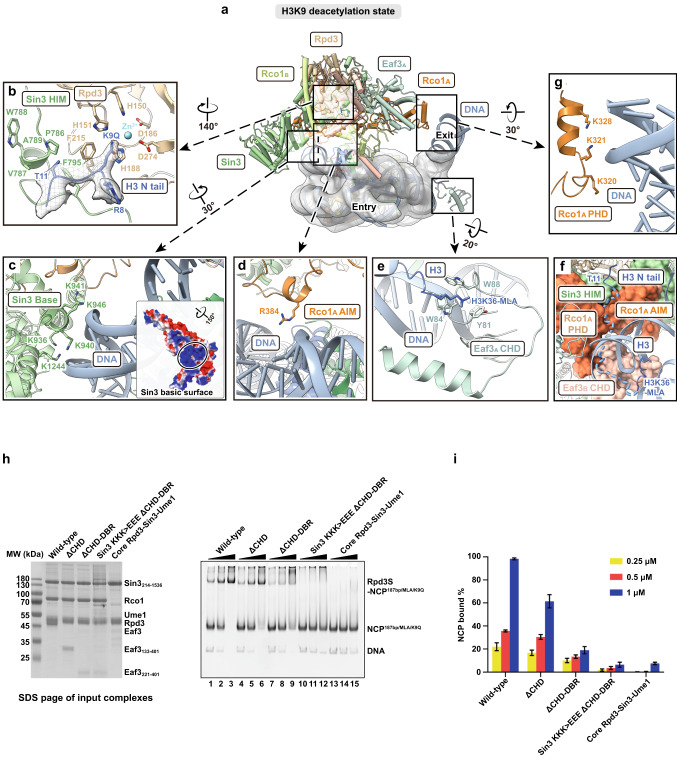
Details of Rpd3S bound to the nucleosome in the H3K9 deacetylation state. **a** An overview of the Rpd3S–NCP^187bp/MLA/K9Q^ complex highlighting the interaction interfaces. The complex was rotated by ~90° from its dyad view in Fig. 1c along the *y* axis. **b** Close view showing the H3 N-terminal tail containing K9Q accommodated by the active site of Rpd3, guided by the Sin3^HIM^ trough and coordinated by the Zn^2+^ ion for catalysis. **c** Interaction of the Sin3 basic surface with the SHL + 2 DNA in this state. The electrostatic surface potential (–/+3.0) is shown on the Sin3 basic surface. **d** Interaction of the Rco1_A_ AIM residue R384 with DNA. **e** Interaction between the Eaf3_A_ CHD with the H3K36MLA tail and the SHL + 7 DNA. The H3K36MLA extends into CHD’s aromatic pocket. This interaction also applies to the Eaf3_B_ CHD. **f** The flexible H3 N-terminal tail (dashed line) is guided by a passage created by the Eaf3_B_ CHD, Rco1_A_, and Sin3 HIM, that leads to the active site of Rpd3. **g** Interaction of MRG_A_-PHD_A_ arm with DNA is coordinated by Rco1_A_ K320/321/328. **h** Interaction between Rpd3S and NCP requires both the CHD and the conserved Sin3 basic surface. EMSA showing Rpd3S binding to the NCP. Rpd3S and its variants (0.25 μM, 0.5 μM, 1 μM) were titrated to interact with 0.7 μM NCP^187bp/MLA/K9Q^. ΔCHD stands for CHD deletion. ΔCHD-DBR stands for the deletion of CHD and DNA-binding region. Sin3 KKK > EEE stands for K936E/K941E/K946E mutations. Core stands for Rpd3–Sin3–Ume1 ternary complex. SDS-PAGE of input complexes was displayed to indicate the integrity of each complex. **i** Statistics for 4 replicate experiments of **h**.

3D classification of the Rpd3S–NCP^187bp/MLA^ complex results in one overall conformation containing three classes, in which the Rpd3S broadly engages the NCP at the SHL + 2 position with slightly different local positionings. Further local refinement of Rpd3S and NCP density allowed us to obtain high-resolution maps of Rpd3S–NCP^187bp/MLA^ complex with the Rpd3S complex at 2.8–3.1 Å and NCP at 2.6–2.7 Å resolution (Supplementary information, Figs. S7a, S10 and Table S1). We rationalize that the local differential positionings of Rpd3S were due to the absence of an acetylated histone substrate and that the plasticity allows the HDAC to sample H3/H4 histone tails with different modifications. To test this hypothesis, we further introduced an acetyl-lysine analog to mimic a previously known Rpd3S target, acetylated H3K9 (Supplementary information, Fig. S8a, b),^22,39^ by mutating the lysine-9 residue to glutamine (K9Q)^26,40^ with the aim to lock Rpd3S at a definitive position at SHL + 2. 3D classification and further local refinement of the Rpd3S–NCP^187bp/MLA/K9Q^ complex indeed results in one single SHL + 2 class with the Rpd3S complex at 2.65 Å and NCP at 2.55 Å resolution (Figs. 1b, c, 2a; Supplementary information, Figs. S11, S14 and Table S1). We named the 3D classes of Rpd3S–NCP^187bp/MLA^ at the SHL + 2 positions collectively as the H3/H4 deacetylation states of the Rpd3S–NCP complex, and we termed the K9Q-locked class as the H3K9 deacetylation state. These high-resolution maps of Rpd3S at the SHL + 2 positions enabled de novo model building and allowed structural comparison with the apo Rpd3S structure (Fig. 1d; Supplementary information, Table S1).

Within the H3/H4 deacetylation states, the active site of Rpd3 exhibits positional shifts of approximately 10–15 Å (Supplementary information, Fig. S7b). The Rpd3 active site is ~33 Å away from the H4 N-terminal residue Leu22 (Supplementary information, Fig. S7c). A peptide of 9 residues could, in theory, cover this distance, which suggests that Rpd3S is able to target the H4 N-terminal tail from this location (Supplementary information, Fig. S7c). Significantly, within one of the three H3/H4 deacetylation states, designated as class 3, the H4 N-terminal main chain exhibits a distinctive trajectory leading towards the active site of Rpd3, unlike the other two H3/H4 deacetylation states (Supplementary information, Fig. S7d). Notably, weak density from a putatively unmodified histone tail can be observed near the Rpd3 active site in these H3/H4 deacetylation states (Supplementary information, Fig. S7e, f). This supports the hypothesis that Rpd3S can sample histone tails at the SHL + 2 position for deacetylation in the absence of the H3K9Q substrate mimic.

**Fig. 3:**
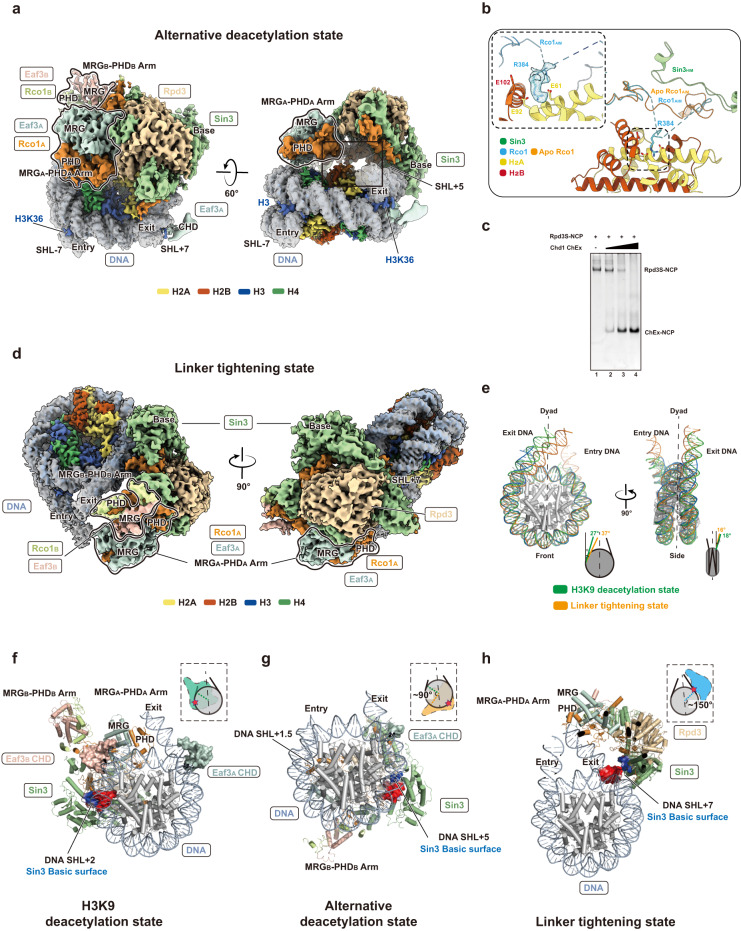
Details of Rpd3S bound to the nucleosome in the alternative deacetylation state and linker tightening state. **a** Top view (left) and dyad view (right) of the cryo-EM map of the Rpd3S–nucleosome complex at its alternative deacetylation state. Two MRG-PHD arms are illustrated with bold contours. In this state, the Sin3 basic surface binds to the SHL + 5 DNA. **b** Interaction of the R384 anchor of Rco1_A_ AIM with the H2A/H2B acidic patch. The local EM density of R384 anchor is closely viewed. The Rco1_A_ AIM changes the conformation to extend its interaction with the acidic patch. **c** ChEx competition assay confirmed that Rpd3S interacts with the acidic patch. 100 nM Rpd3S–NCP in 6 μL reaction system was shown in lane 1 as the control. The titration of Chd1 ChEx (62.5, 125, and 250 μM) was used to interact with Rpd3S–NCP for 30 min. Increased ChEx concentrations can out-compete Rpd3S away from nucleosome, resulting in the formation of the ChEx–NCP complex (lanes 2 to 4). **d** Top view (left) and side view (right) of the cryo-EM map of the Rpd3S–nucleosome complex at its linker tightening state. In this state, the Sin3 basic surface binds to the SHL + 7 DNA. Both MRG_A_-PHD_A_ and MRG_B_-PHD_B_ arms interact with the entry and exit DNA linkers, respectively. **e** Comparison of the exit DNA linker angles between the H3K9 deacetylation state and linker tightening state. **f**–**h** Side-by-side comparison in bottom view of Rpd3S in complex with NCP in three states. The Sin3 basic surface (blue) and corresponding interacting DNA (red) are shown in electrostatic potential surfaces. The Eaf3_A/B_ CHDs are shown as surfaces. MRG-PHD arms are labeled.

In all H3/H4 and the locked H3K9 deacetylation states, Rpd3S binds to SHL + 2 of the NCP via a conserved Sin3 basic surface while the MRG_A_-PHD_A_ arm binds to the phosphodiester backbone of the exiting DNA linker via Rco1_A_ K320/K321/K328 (Figs. 1d, 2c, g, 3f). This structural observation is consistent with a previous study showing that mutations in Rco1 PHD1 (known as PHD in our study) lead to the initiation of cryptic transcription.^10^ Because mutations of Rco1 PHD will likely disrupt the structural integrity of the MRG_A_-PHD_A_ arm, the ability to bind to the DNA linker is also compromised. Both H3K36MLA tails protrude through the gyres’ minor grooves at SHL – 7 and SHL + 7, fitting into the conserved aromatic pockets of the CHDs from Eaf3_A_ and Eaf3_B_, which engages the local nucleosomal DNA at cross-gyre manners (Figs. 1c, d, 2a, e, 3f). The Sin3 basic surface and the CHDs are essential in binding NCP, as combining mutations on key Sin3 conserved basic residues K936E/K941E/K946E (Supplementary information, Fig. S4c) and the deletion of the Eaf3 CHDs result in a dramatic reduction in the Rpd3S–NCP complex formation in electrophoretic mobility shift assay (EMSA) (Fig. 2h, i). The deletion of the CHD or the combined deletion of both the CHD and the DNA-binding region (DBR) of Eaf3 do not affect the integrity of the complex as both Rpd3S^ΔCHD^ and Rpd3S^ΔCHD-DBR^ can still be purified (Fig. 2h). Therefore, the reduction in NCP binding is not due to the disruption of the structural integrity of Rpd3S.

In the H3K9 deacetylation state, in which the deacetylation activity of Rpd3S was confirmed by the nucleosomal H3K9ac deacetylation assay (Supplementary information, Fig. S8), the active site of the Rpd3 subunit is occupied by the acetyl-lysine analog H3K9Q, whose C-terminal region lies on a hydrophobic trough formed between the conserved underwound helix of P786/V787/W788/A789 and F795 of Sin3 HIM (Fig. 2b). The Rpd3 active site is ~58 Å away from the H3K36MLA residue at SHL – 7 (Fig. 2f). Considering a peptide of 17–25 residues could span across this distance, Rpd3S may be able to target the H3 N terminus from this location (Fig. 2f). Rco1_A_ AIM, which adopts loop conformation in apo Rpd3S, changed to a helical segment at residues R384/Q385/L386/F387 and binds to the phosphodiester backbone of the NCP via R384 (Fig. 2d; Supplementary information, Fig. S5).

Overall, our structures suggest that Rpd3S primarily targets H3/H4 histone tails through a concerted mechanism involving the coordinated bindings of the Sin3 basic surface to core nucleosomal DNA at SHL + 2, Eaf3 CHDs to H3K36MLA, and the MRG_A_-PHD_A_ arm to the exit DNA linker. We propose that the H3/H4 and H3K9 deacetylation states capture snapshots of Rpd3S being in the process of sampling and targeting acetylated H3/H4 on the nucleosome.

### Removal of DNA entry linker frees Rpd3S for alternative deacetylation

It was reported that chromatin remodelers fine-tune nucleosome spacing to control Rpd3S deacetylation activity.^41^ We rationalized that the H3/H4 deacetylation states obtained were due to the combined interactions between DNA linker and H3K36MLA with Rpd3S. To mimic the DNA linker undergoing remodeling in the wake of RNAPII, we removed the 20 bp DNA entry linker from the NCP (NCP^167bp/MLA^) to reconstitute a complex with Rpd3S. 3D classification of the Rpd3S–NCP^167bp/MLA^ complex results in two predominant Rpd3S binding states, one of which resembles the H3/H4 deacetylation states at SHL + 2 and the other at SHL + 5 we termed the alternative deacetylation state since Rpd3S could deacetylate multiple histone tails including H2A and H2B^18,19^ (Supplementary information, Figs. S12, S14 and Table S1).

In the alternative deacetylation state, Rpd3S is freed from being constrained by the Eaf3_B_-entry linker interaction and rotates by ~90° along the plane of the nucleosomal disc relative to the H3/H4 deacetylation states to bind to the top side of the NCP via multiple contacts (Fig. 3a). On nucleosomal DNA, the conserved Sin3 basic surface and the MRG_A_-PHD_A_ arm bind to the phosphodiester backbone of the SHL + 5 and SHL + 1.5 of the NCP, respectively (Fig. 3g). The CHD of Eaf3_A_ continues to interact with the H3K36MLA tails and engages the nucleosomal DNA at SHL + 7 (Fig. 3a, g). At SHL – 7, the Eaf3_B_ CHD is no longer visible, although the density of the H3K36MLA tail is still present (Fig. 3a). Together with the EMSA data showing Eaf3 CHDs are essential for optimal binding of NCP by Rpd3S (Fig. 2h), we propose that the interaction between CHDs and H3K36me3 is one of the key factors in maintaining Rpd3S on the nucleosomal disc at certain positions in order to sample and target multiple histone tails.

On Rpd3S, Rco1_A_ AIM leaves HIM and becomes largely invisible, while the conserved R384 fits into an acidic pocket created by H2A (E61/E92) and H2B (E102) at the acidic patch (Fig. 3b), hence the naming of the AIM. The interaction between Rpd3S and the acidic patch is further confirmed by the finding that a Chd1 ChEx peptide, a known acidic-patch binding peptide,^42^ is able to compete Rpd3S away from the NCP (Fig. 3c). Cryo-EM image processing of Rpd3S–NCP^167bp/MLA^ reveals that the number of particles with Rpd3S binding to the SHL + 2 position is four times more than that at the SHL + 5 position (Supplementary information, Fig. S12). Therefore, it is likely that Rpd3S has a higher affinity towards the NCP at SHL + 2 and that this position is the first point of Rpd3S contact on the H3K36 trimethylated NCP. Since Ume1 homologue mammalian RbAp46/48 and *Drosophila* Nurf55/P55 are the histone chaperone of H3 and H4,^43–45^ Rpd3S could potentially utilize the Ume1 subunit to assemble H3/H4 and target their histone tails at SHL + 2 position prior to transitioning to the alternative deacetylation state at SHL + 5 where it could further target other proximal histone tails such as H2A/H2B.^18,19,21^

### Rpd3S tightens DNA linkers post deacetylation

Having understood the structural mechanism of deacetylation by Rpd3S, we created an Rpd3S–NCP^187bp^ complex that mimics a stage when the nucleosome is deacetylated and Rpd3S is no longer activated by methylation. 3D classification of the Rpd3S–NCP^187bp^ complex results in two Rpd3S binding states (Supplementary information, Figs. S13, S14 and Table S1). One is identical to the SHL + 2 state and another novel state that we termed the linker tightening state (Fig. 3d). In the linker tightening state, Rpd3S rotates around the nucleosomal DNA axis by ~150° with the Sin3 conserved basic surface binding to the nucleosomal DNA at the SHL + 7 position (Fig. 3d). This rotation makes the Rpd3S main body leaving the nucleosomal disc entirely, with MRG_A_-PHD_A_ and MRG_B_-PHD_B_ arms interacting with the entry and exit DNA linkers (Fig. 3d, h). The position of Rpd3S in the linker tightening state, which is created without H3K36MLA, further reinforces the notion that the interaction between Eaf3 CHDs and H3K36MLA is the key factor in maintaining Rpd3S on the nucleosomal disc and hence the ability of Rpd3S to sample and target histone tails within proximity. Notably, by measuring the angle between the linker and the dyad axis in the planes parallel (α) and perpendicular (β) to the nucleosomal disc plane, we observed that the exit DNA linker bends with an α angle of 37° in the linker tightening state compared to 27° in H3K9 deacetylation state (Fig. 3e). This indicates that Rpd3S further tightens the exit DNA linker post deacetylation.

It is generally accepted that the histone–DNA interactions at the nucleosomal entry/exit DNA regions are weaker compared to those at core nucleosomal DNA.^46^ The entry/exit regions are often the binding sites for transcription factors,^47–49^ which unwrap the nucleosomal DNA for RNAPII passage. Collectively, our results indicate that following deacetylation and absence of constraints from H3K36me3, Rpd3S disengages from the nucleosomal disc while maintaining its interactions with the DNA linkers, which are in turn tightened. The Rpd3S-mediated tightening of the DNA linker could potentially serve as a mechanism that counteracts the binding of transcription factors to nucleosomes at cryptic start sites for repressing cryptic transcription.

### Rpd3S acts in concert with Hho1 for NCP engagement

**Fig. 4:**
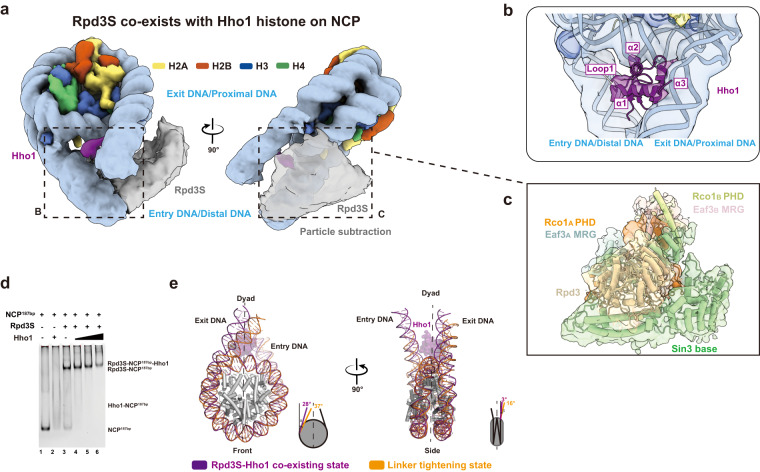
Rpd3S concurrently participates in NCP engagement along with Hho1. **a** Top view (left) and side view (right) of the cryo-EM map of Rpd3S co-existing with Hho1 linker histone on the NCP. **b** Focus refinement of potential Hho1 density with nucleosome revealed clear helical densities that can accommodate the Hho1 globular domain structure. **c** Particle subtraction of potential Rpd3S density and ab-initial reconstruction revealed an ~ 6 Å map that can unambiguously accommodate the apo Rpd3S complex structure. **d** EMSA showed Rpd3S co-existing with Hho1 in NCP^187bp^. 150 nM NCP^187bp^ without or with 37.5 μM Hho1 in 7 μL reaction system were shown in lanes 1 to 2 as controls; the Hho1 titration (0 μM, 12.5 μM, 37.5 μM, 112.5 μM, lanes 3 to 6) promoted the bands of Rpd3S–NCP^187bp^ to shift up, resulting in the formation of Rpd3S–NCP^187bp^–Hho1. **e** Comparison of exit DNA linker angles between the linker tightening state and the Hho1 co-existing state.

**Fig. 5:**
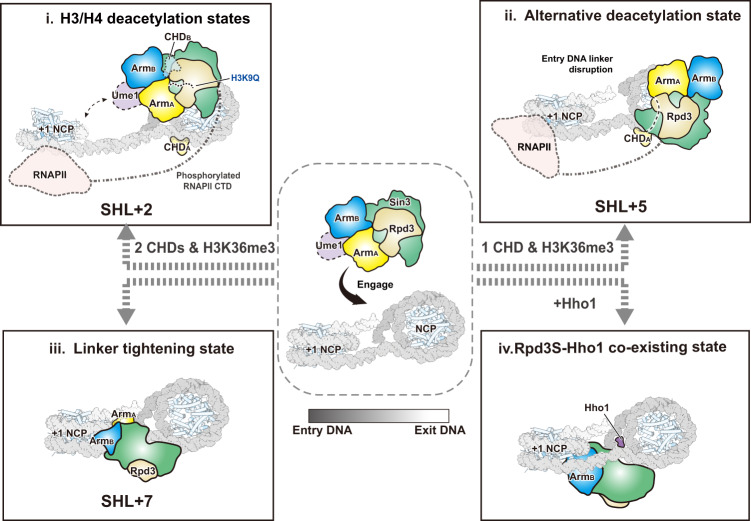
A cartoon depicting Rpd3S recognizes nucleosome substrates in multiple states for deacetylation and DNA linkers reconfiguration. i Rpd3S recognizes and deacetylates the H3/H4 N termini. ii Rpd3S moves to SHL + 5 where it can potentially deacetylate other histones such as H2A/H2B. iii Rpd3S leaves the NCP disc and utilizes its DNA interaction surfaces to tighten the extra-nucleosomal DNA post-deacetylation, implying that Rpd3S may counteract pioneer factors/transcription factors to repress cryptic transcription. iv In certain regions where Hho1 linker histones are required for gene silencing, Rpd3S could act in concert with Hho1 to engage the NCP and facilitate the subsequent nucleosome array compaction.

H1 linker histone has been shown to engage chromatin post-deacetylation to facilitate the formation of a higher-ordered nucleosome array after transcription.^24–26^ Chromatin remodelers, such as Chd1 and Isw1, can fine-tune the histone deacetylation activity of Rpd3S by altering the nucleosomal spacing and compete with Hho1 to bind to nucleosomes in vivo.^41,50,51^ Furthermore, Hho1 utilizes its two globular domains to interact with di-nucleosome,^51^ which is a preferred substrate of Rpd3S.^20,41^ To explore the possibility of Rpd3S poising the NCP for H1 engagement, we recombinantly purified the globular domain of the Hho1 and reconstituted an Rpd3S–NCP^187bp^–Hho1 complex for structure determination (Supplementary information, Fig. S13 and Table S1). 3D classification reveals that 15.2% of particles contain the putative Hho1 density (Supplementary information, Fig. S13). This allows the reconstruction of a consensus map of 6.2 Å containing both the Rpd3S and Hho1 densities (Fig. 4a). Further focus refinement of the Hho1 density revealed clear helical densities, in which Hho1 globular domain can be fitted (Fig. 4a, b). Hho1 exhibits canonical dyad binding with its loop 1 and helix 3 coordinating the two DNA linkers while helix 2 contacts the dyad (Fig. 4b). The Rpd3S in the consensus map exhibits substantial flexibility, resulting in a fragmented density that rotates further away from the NCP body towards the entry DNA linker. To confirm the fragmented density being Rpd3S, we performed density subtraction and subsequent ab-initial reconstruction, which yielded a map of 6 Å (Supplementary information, Fig. S13). This map can unambiguously accommodate the Rpd3S complex structure, thus confirming the co-existence of Rpd3S and Hho1 on the NCP (Fig. 4c). The co-existence of the Rpd3S and Hho1 on NCP^187bp^ was further confirmed by the EMSA (Fig. 4d). In order to accommodate Hho1, the exit/proximal linker adopts a characteristic bend with a β angle of ~3° (Fig. 4e), which reminisces those of linker histone H1–NCP structures.^52–55^ Taken together, our combined structural data demonstrate that Rpd3S samples the NCP at multiple nucleosomal superhelical locations to deacetylate its histone substrates and acts in concert with Hho1 to engage the NCP^26^ (Fig. 5).

## Discussion

In this study, we recapitulate the dynamic states of nucleosome deacetylation and DNA linker tightening mediated by Rpd3S (Fig. 5). Rpd3S is recruited by the phosphorylated CTD of RNAPII, leading it to travel with RNAPII.^6,7^ Rpd3S utilizes the conserved Sin3 basic surface to bind to the nucleosomal DNA positions at SHL + 2, SHL + 5, and SHL + 7, under the conditions involving different H3K36me3–CHD interactions and DNA linker interactions. Based on these states, we propose a working model of Rpd3S probing and engaging with multiple nucleosomal positions, thus providing a conceptual framework for understanding the complex’s multifaceted engagement with nucleosomes. Additionally, inspired by recent studies that elucidated the progression of RNAPII through nucleosomes,^56,57^ the H3K36me3- and DNA linker-mediated nucleosomal positionings may also imply that Rpd3S could navigate through the NCP in a way similar to the superhelical passage taken by the RNAPII on NCP.^56,57^ However, the actual dynamic behavior of the complex still requires further investigation.

During the initial engagement, Rpd3S may be guided by its interactions with H3K36me3 and the DNA linkers to bind to the SHL + 2 position of the nucleosomal DNA. The flexible H3 N-terminal tail protruded from the gyres’ minor groove at SHL – 7 is putatively guided by a passage created by the Eaf3_B_ CHD, Rco1_A_, and Sin3 HIM that leads to the active site of Rpd3 for the deacetylation (Fig. 2f). The H3K9 deacetylation state obviously represents a snapshot of Rpd3S deacetylating H3K9ac as the introduction of the acetyl-lysine mimic H3K9Q locks the Rpd3S–NCP^187bp/MLA/K9Q^ complex in a single conformation. In this state, the MRG_A_-PHD_A_ arm binds to the exiting DNA linker at SHL + 7, likely facilitating the initial DNA linker tightening (Fig. 3e).

Once the nucleosome spacing is fine-tuned by chromatin remodelers such as Chd1 and Isw1,^41,58^ Rpd3S may lose its stable interaction with the DNA linker and H3K36me3. Without the constraint by the Eaf3_B_ CHD at SHL – 7, Rpd3S could gain the freedom to move away from its SHL + 2 position and reposition itself at SHL + 5 on the top side of the NCP (Fig. 5). H2A/H2B could be the potential target of this state due to the local proximity. The transition to this alternative deacetylation state is exhibited by the Rpd3S–NCP^167bp/MLA^ complex, which is made possible by the Sin3 basic surface maintaining Rpd3S on the nucleosomal DNA track. The repositioning at SHL + 5 is likely guided by Rco1_A_ AIM’s interaction with the acidic patch and Eaf3_A_ CHD interaction with H3K36MLA at SHL + 7 (Fig. 3a, g).

In the Rpd3S–NCP^187bp^ complex, the absence of H3K36MLA further removes the constraints on Rpd3S and allows it to leave the NCP disc entirely (Fig. 5). In this linker tightening state, Rpd3S binds to the SHL + 7 position via the conserved Sin3 basic surface, while the MRG_A_-PHD_A_ and MRG_B_-PHD_B_ arms interact and tighten the DNA linkers. The further tightening could be a precautionary step to prevent the cryptic binding of transcription factors at the nucleosomal entry/exit DNA regions and thus suppressing spurious intragenic transcription.

Rpd3S is allosterically activated by H3K36me3.^36^ By comparing the distinct nucleosomal locations of Rpd3S in the H3/H4 deacetylation states, the alternative deacetylation state, and the linker tightening state, we suggest that apart from the recognition of H3K36me3 by Eaf3 CHDs, the binding of MRG-PHD arms to the DNA linkers is also likely to be a factor in nucleosome recognition by Rpd3S. A previous study showed that mutations in Rco1 PHD1 (known as PHD in our study) would result in cryptic transcription initiation.^10^ As our structures reveal that PHD binds to Eaf3 MRG, PHD mutations will most likely disrupt the structural integrity of the MRG-PHD arms and affect their interaction with DNA linkers. Therefore, we propose that the combined engagement of H3K36me3 and DNA linkers cooperatively activates Rpd3S by restraining it to certain positions on the NCP where the active site of Rpd3 is close enough to sample different histone tails for deacetylation.

During the revision of this manuscript, Guan et al. reported the cryo-EM structures of Rpd3S–NCP in the ‘close’ and ‘loose’ states.^59^ These states are structurally equivalent to the H3/H4 deacetylation states described in our study. The investigation of the alternative deacetylation state and linker tightening state in our study provides additional insights into the diverse modes of Rpd3S engaging the nucleosomal substrate and outlines a putative working mechanism, which has not been proposed before.

Previously, genome-wide analysis revealed that there was an inverse correlation between linker histone occupancy and histone acetylation.^26^ Furthermore, an *rpd3Δ* mutant rescued the growth defect caused by Hho1 overexpression in a synthetic dosage screen.^26^ These results suggest that Rpd3 could set nucleosomes up for Hho1 binding in cells by deacetylating histone tails. Due to the fine-tuning of the linker angles by Rpd3S and the potential optimization of nucleosomal spacing by chromatin remodelers,^41,50^ a favorable environment can be created for the engagement of Hho1 linker histone. Our Rpd3S–NCP^187*bp*^–Hho1 structure, which exhibits a canonical DNA linker conformation of an H1–NCP complex,^53,55^ unites our structural observations with the abovementioned in vivo studies and compels us to propose a novel idea that Rpd3S acts in concert with Hho1 to engage the NCP by reconfiguring DNA linker angles. This Rpd3S–Hho1 co-existing state on NCP could be an intermediate state (Fig. 5) of Rpd3S moving towards the next cycle of histone deacetylation while promoting transcription repression mediated by Hho1 in certain genes.^24,27,50^

## Materials and methods

### Preparation and purification of Rpd3S complex

The coding sequences of Rpd3S subunits (*Rpd3*, *Sin3*(214–1536), *Ume1*, *Rco1*, and *Eaf3*) were amplified by PCR respectively and were cloned into modified pFBDM vectors.^60^ The double-StrepII tag was engineered at the N terminus of Sin3 and C terminus of Rco1 for affinity purification. The viruses containing five-subunit genes of Rpd3S were mixed to infect High Five insect cells and co-expressed for 54 h. The insect cells were pelleted and lysed in buffer A (25 mM Tris-HCl pH 7.5, 300 mM NaCl, 0.5 mM TCEP, 10% glycerol, 1 mM PMSF, Protease inhibitor cocktail and Supernuclease) via the high-pressure homogenizer. The lysate was spun at 22,000 rpm for 1 h. After the binding and washing to the streptavidin column, the protein complex was eluted in buffer A with 25 mM Desthiobiotin. The fractions containing Rpd3S were diluted to buffer B (10 mM Tris-HCl pH 7.5, 80 mM NaCl, 10% glycerol, 0.5 mM TCEP) and then purified by anion-exchange chromatography with Resource Q column (GE Healthcare) and eluted with a gradient 0%–100% buffer C (10 mM Tris-HCl pH 7.5, 1 M NaCl, 10% glycerol, 0.5 mM TCEP). Finally, the protein complex was further purified by gel filtration in buffer D (25 mM HEPES pH 7.5, 150 mM NaCl, and 0.5 mM TCEP). The peak fractions from gel filtration were concentrated and the aliquots were stored at −80 °C.

### Preparation and purification of histone octamer

The modified vector pETDuet-1-*Xenopus laevis* co-expresses the histones in octamer form using a polycistronic approach.^61^ *Escherichia coli* Rosetta (DE3) cells containing this vector were induced with 0.4 mM IPTG when the OD_600_ reached 0.4–0.5. The culture was further shaken at 170 rpm at 37 °C for 20 h. These bacterial pellets were lysed in buffer (20 mM Tris pH 8.0, 500 mM NaCl, 0.1 mM EDTA, 0.5 mM TCEP), and the supernatant was loaded into the Heparin affinity column (Cytiva). The column was washed with 500 mM NaCl buffer and slowly eluted with a salt gradient from 500 mM to 2 M NaCl. Since these recombinant histones were wrapped with a lot of *E. coli* DNA, a gel filtration with buffer containing 2 M salt (20 mM Tris pH 8.0, 2 M NaCl, 0.5 mM EDTA, 0.5 mM TCEP) was used to remove the endogenous DNA. The histone octamer was eluted as a single peak, although a few fragile protein peaks were also found.

### Preparation of H3K36MLA containing histone octamer

Histone H3 C110 was mutated to alanine and later H3K36 was mutated to cysteine. Histone octamer containing H3 with these double mutations was expressed and purified as wild-type histone octamer. The H3K36C of the octamer was modified by the installation of MLA as previously described.^37^ (2-bromoethyl) trimethyl ammonium bromide reagent was used to generate histone octamer containing-H3K36me3 (MLA). To generate the acetyl-mimetic MLA mutant, lysine (K) 9 of H3 was mutated to glutamine (Q) within the C110A/K36C mutant and later MLAs were installed.

### Preparation of Rco1-deleted strain and extraction of endogenous histone octamer

The *Saccharomyces cerevisiae* strain used in this study is derived from BY4741.^62^ Rco1 was deleted using the rapamycin-mediated “anchor away” technique.^63^ To extract the endogenous histone octamer, the Rco1-deleted strain was grown in YPD media at 30 °C at 220 rpm shaking until the OD_600_ reached 0.8. The purification of endogenous histone octamer was the same as the recombinant histone octamer.

### Preparation of Hho1 protein

The expression vector of Hho1 globular domain, pETDuet-1-His-TEV-Hho1 (residues 41–117), was synthesized. *E. coli* BL21(DE3) cells containing this vector were induced with 0.4 mM IPTG when the OD_600_ reached 0.4–0.8 and further shaken at 170 rpm at 37 °C for 6 h. This Hho1 protein was purified initially using the His-tag affinity chromatography column, and His-tag was removed using Tobacco etch virus protease (TEV). Similar to the histone octamer purification, one round of gel filtration in 2 M salt was used for removing bacterial endogenous DNA, followed by a second round of gel filtration in a low salt buffer (25 mM HEPES, pH 7.5, 150 mM NaCl, and 0.5 mM TCEP). The peak fractions of Hho1 were concentrated and the aliquots were stored at −80 °C.

### Preparation of nucleosome

The ‘double bag’ dialysis approach was used to prepare the nucleosome.^64^ DNA was resuspended in the high-salt buffer (20 mM Tris pH 8.0, 2 M NaCl, 0.5 mM EDTA, 0.5 mM TCEP) and mixed with the histone octamer which was in the same high-salt buffer, and the molar ratio of histone octamer to DNA was 1.1 to 1. Dialysis buttons made from an Eppendorf tube lid holding 0.2 mL of the histone octamer-DNA mixture were put inside a dialysis bag containing 50 mL of the high-salt buffer. The dialysis was performed overnight at 4 °C using 1 L buffer containing 20 mM Tris pH 8.0, 1 M NaCl, 0.5 mM EDTA, and 0.5 mM TCEP. After 12 h, the dialysis bag holding 50 mL of 1 M salt buffer and the dialysis buttons were submerged in 1 L low-salt buffer (20 mM Tris pH 8.0, 50 mM NaCl, 0.5 mM EDTA, 0.5 mM TCEP) and was dialyzed for 5–6 h. Finally, only the dialysis buttons were further dialyzed in the low-salt buffer for 3–4 h.

### Analytical gel filtration chromatography

Analytical gel filtration chromatography was performed on an ÄKTA Pure Protein Purification System (Cytiva). Protein samples (10–50 μM in 300 μL) were injected into a Superose 6 increase 10/300 GL column (Cytiva) that was running with gel filtration buffer (10 mM HEPES pH 7.5, 150 mM NaCl, 0.5 mM TCEP).

### Size exclusion chromatography-MALS (SEC-MALS)

100 μL Rpd3S sample (40 μM) was loaded onto a Superdex 200 5/150 column (Cytiva) with a buffer containing 20 mM Tris-HCl pH 7.5, 150 mM NaCl. A static light-scattering detector and a differential refractive index detector (Wyatt) were connected to the analytical gel filtration chromatography system. Data were analyzed with ASTRA7 provided by Wyatt.

### EMSA

The samples were loaded and run on a 4.5% native PAGE gel for 100 min at 70 Volts with 0.5× TBE buffer at 4 °C. The gels were stained in 10 mL 0.5× TBE buffer with 1–2 μL of ethidium bromide for 10 min at room temperature. The gels were subsequently washed with TBE buffer and imaged using the ChemiDoc (Bio-Rad) in UV mode.

### Sample preparation of EM

The gradient master was used to prepare the gradient by mixing the top solution and bottom solution. The top solution (20 mM HEPES pH 7.5, 50 mM NaCl, 10% glycerol) and bottom solution (20 mM HEPES pH 7.5, 50 mM NaCl, 30% glycerol, 0.125% glutaraldehyde) was prepared. 6 mL top solution was loaded into the 13 mL centrifuge tube (Beckman), and 6 mL bottom solution was then slowly injected into the tube bottom using a syringe with a long needle. The centrifuge tubes were covered completely. And then the gradient master was used to roll the centrifuge tubes with different programs that have different speeds and angles. The protein complex samples (150 μL about 10 mg/mL) were added above the solution level of centrifuge tubes and spun down for 14 h at 35,000 rpm and 4 °C in the SW41-Ti centrifuge rotor (Beckman). The independent fraction separator gently sucked out around 30 fractions from each tube. SDS-PAGE gels or native gels were used to detect these fractions. The cryo-EM grids (Quantifoil, Au, R1.2/R1.3 300 mesh) were treated in a glow discharge system (GloQube) and the cryo-EM samples were prepared by using the vitrobot (Thermo Fisher Scientific). In the environment of 100% humidity and at 4 °C, 3.5 μL samples were added to the grids, and the girds were blotted for 2 s with force 4 and then inserted into liquid ethane for quick freezing. The girds were screened or stored in liquid nitrogen.

### Negative-stain EM data collection

The copper grids were prepared with a glow discharge, usually 15 mA, negative charge, for 60 s. 3 μL of protein sample was loaded onto the copper girds for 1 min. The excess solution was removed with filter papers and the grids were washed with two drops of ddH_2_O. The grids were stained with two drops of uranium formate, staining for 1 min on the second drop. Excess uranium formate was removed and the grids were air-dried for 10 min. Finally, the micrographs were taken using a 120 kV Tecnai T12 transmission electron microscope (FEI).

### Cryo-EM data collection and image processing

The apo Rpd3S dataset was collected by 200 kV Talos Arctica electron microscope (Thermo Fisher Scientific) using the Serial EM software for automated collection. The images were recorded by a K3 summit direct detector at a nominal magnification of 45,000× in the super-resolution counting mode, which resulted in a super-resolution pixel size of 0.44 Å. The micrographs were fractionated into 27 frames and used a −0.8 μm to –2.2 μm defocus range set with an electron dose rate of 30 e^–^/pix2/s. The raw movies used MotionCor2 in RELION software which aligned with 5 by 5 patches and binned 2-fold to a calibrated pixel size of 0.88 Å/pix. And then, the contrast transfer function was estimated by Gctf,^65,66^ and particles were picked by WARP software.^67^ Several rounds of 2D classification were performed in RELION to discard poorly averaged particles. After generating an initial model in an ab-initial job at CryoSPARC,^68^ the cleaned particles were returned to RELION for 3D classification. Particles from the best 3D class were selected and subjected to 3D auto-refine to reach a higher resolution.

The Rpd3S–NCP datasets were collected by 300 kV Titan Krios electron microscope (Thermo Fisher Scientific) and automatically collected using the EPU software. Movies were recorded by Falcon4 direct electron detector equipped with a SelectrisX energy filter with a 10 eV slit width. In electron event representation (EER) mode, movies were recorded at a nominal magnification of 165,000× with a raw pixel size of 0.71 Å on the image plane. The movies were recorded in a –0.8 μm to −2.2 μm defocus range, with an electron dose rate of 26 e^–^/Å^2^/s and a total dose of 50 e^–^/Å^2^. All the EER movies were processed by CryoSPARC software, and CryoSPARC Live preprocessed the initial motion correction and CTF estimate. After several rounds of 2D classification, the average particles from the good class were submitted to ab-initial. The best initial volume map was selected to further clean by heterogeneous refinement and generate a final global map at homogenous refinement. The volume was split into two and subjected to local refinement. And then two local volume maps were combined in the model building.

### Model building

For the model building of the apo Rpd3S complex, the initial models of Eaf3–Rco1, Rpd3, and PAH3 of Sin3 were based on the published structures (PDB: 2LKM, 1C3P, 2N2H, and 6XAW), respectively. The other subunit sequences were uploaded to the SWISS-MODEL server for initial model generation.^69^ The initial models were rigidly docked into density maps by PHENIX,^70^ and the subsequent modeling was manually built based on the high-resolution map in COOT.^71^ Structure real-space refinement and flexible fitting were performed with PHENIX. For the model building of Rpd3S–NCP complexes, we generated the initial model from the apo Rpd3S structure and manually adjusted it in COOT. NAMDINATOR was used to adjust the map-to-model fit for low-resolution regions and to reduce clashes between atoms for the overall models.^72^ The nucleosome models were based on the available crystal structure (PDB: 4LD9), and the Hho1 model was built by fitting the previous structure (PDB: 7PFX) and then manually edited in COOT. Different-length DNA chains were modeled from different EM maps, which were low-pass filtered to 6 Å. Finally, methylation modifications and mutations were adjusted in COOT. All above models were subjected to PHENIX for several rounds of real-space refinement and obtained validation finally. PyMOL (https://pymol.org/2/) and UCSF ChimeraX were used for the generation of figures.^73^

### XL-MS

The protein samples were exchanged to HEPES buffer and adjusted the concentration to 1.5 mg/mL. For XL-MS analysis, the purified apo Rpd3S was incubated with 1 mM BS3 (bis(sulfosuccinimidyl)suberate) at room temperature for 30 min. The sample was quenched with 50 mM Tris buffer (the same pH as the HEPES buffer). The sample was frozen in liquid nitrogen and stored at −80 °C. The cross-linked protein was digested first and pre-fractionated by HPLC. After desalting, the sample was submitted to LC-MS/MS analysis. The result was analyzed in pLink2 software and viewed on the xiView.^74^

### Supplementary information

Supplementary information, Fig. S1

Supplementary information, Fig. S2

Supplementary information, Fig. S3

Supplementary information, Fig. S4

Supplementary information, Fig. S5

Supplementary information, Fig. S6

Supplementary information, Fig. S7

Supplementary information, Fig. S8

Supplementary information, Fig. S9

Supplementary information, Fig. S10

Supplementary information, Fig. S11

Supplementary information, Fig. S12

Supplementary information, Fig. S13

Supplementary information, Fig. S14

Supplementary information, Table S1

Supplementary figure legend

## Data Availability

The cryo-EM density maps and corresponding atomic coordinates have been deposited in the Electron Microscopy Data Bank (EMDB) and Protein Data Bank (PDB) under accession numbers EMD-37096 and PDB-8KC7 for the apo Rpd3S complex; EMD-37123 and PDB-8KD3 for the Rpd3S–NCP^187bp/MLA/K9Q^ complex; EMD-37124/PDB-8KD4, EMD-37125/PDB-8KD5 and EMD-37126/PDB-8KD6 for the Rpd3S–NCP^187bp/MLA^ complex class1, 2 and 3, respectively; EMD-37127 and PDB-8KD7 for the Rpd3S–NCP^167bp/MLA^ complex; EMD-37122 and PDB-8KD2 for the Rpd3S–NCP^187bp^ complex.
