# Supplementary material for: Structural basis of nucleosome deacetylation and DNA linker tightening by Rpd3S histone deacetylase complex
Source: Cell Res. 2023 Sep 4;33(10):790–801. doi: 10.1038/s41422-023-00869-1 (PMC10542350; doi:10.1038/s41422-023-00869-1)
Supplement: Supplementary file 1 — Supplementary information, Fig. S1 [file 41422_2023_869_MOESM1_ESM.pdf]

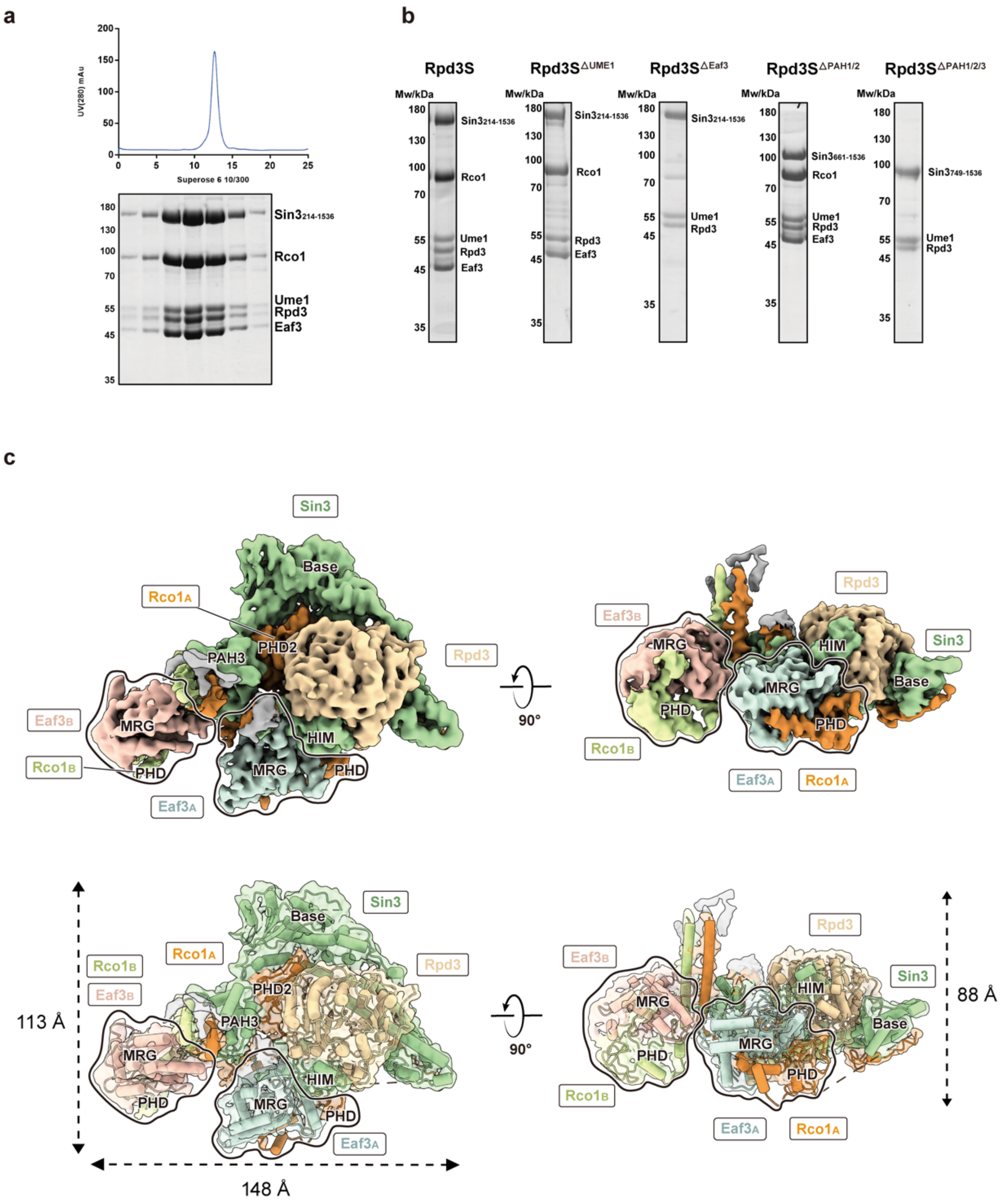

Supplementary information, Fig. S1. Purification and cryo-EM structure of apo Rpd3S. **a,** Rpd3S was purified using gel filtration and the peak fractions were subjected to SDS-PAGE for Coomassie blue staining. **b,** Purified apo Rpd3S and four truncated complexes, Rpd3S$^{\Delta Ume1}$, Rpd3S$^{\Delta Eaf3}$, Rpd3S$^{\Delta PAH1/2}$ and Rpd3S$^{\Delta PAH1/2/3}$. **c,** Structure of apo Rpd3S. Two views of the high-resolution map and ribbon representations of apo Rpd3S. PHD2 domain (residues 416-470) is labelled. The three dimensions of Rpd3S are 148 Å x 113 Å x 88 Å.
