# Supplementary material for: Structural basis of nucleosome deacetylation and DNA linker tightening by Rpd3S histone deacetylase complex
Source: Cell Res. 2023 Sep 4;33(10):790–801. doi: 10.1038/s41422-023-00869-1 (PMC10542350; doi:10.1038/s41422-023-00869-1)
Supplement: Supplementary file 2 — Supplementary information, Fig. S2 [file 41422_2023_869_MOESM2_ESM.pdf]

**a**

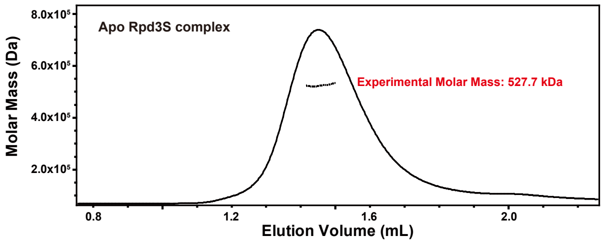


| Subunits | Molar Mass (kDa) | Copy |
|---|---|---|
| $Sin3_{214\text{-}1536}$ | 157.46 | 1 |
| Rpd3 | 48.90 | 1 |
| Rco1 | 83.06 | 2 |
| Eaf3 | 45.20 | 2 |
| Ume1 | 51.02 | 1 |
| Theoretical Molar Mass (kDa) | | 513.90 |

**b**

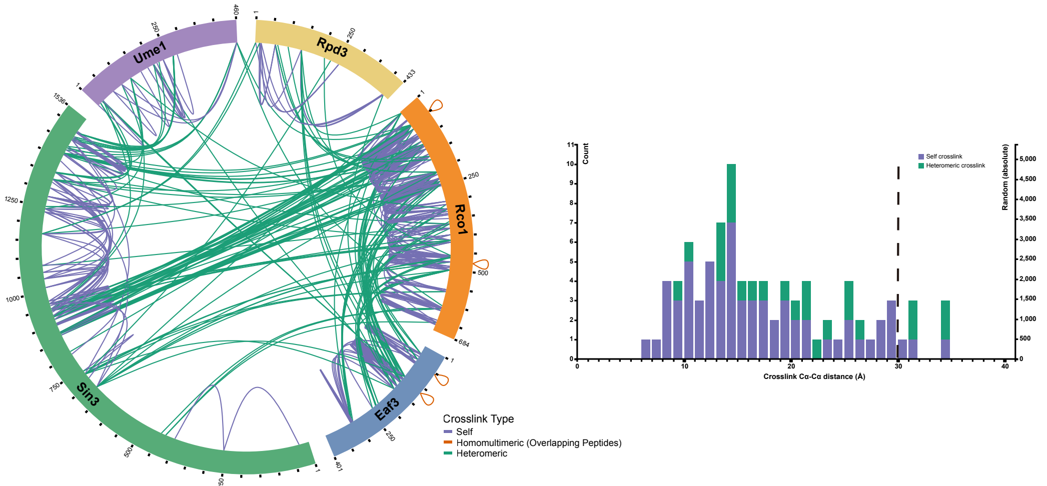


**c**

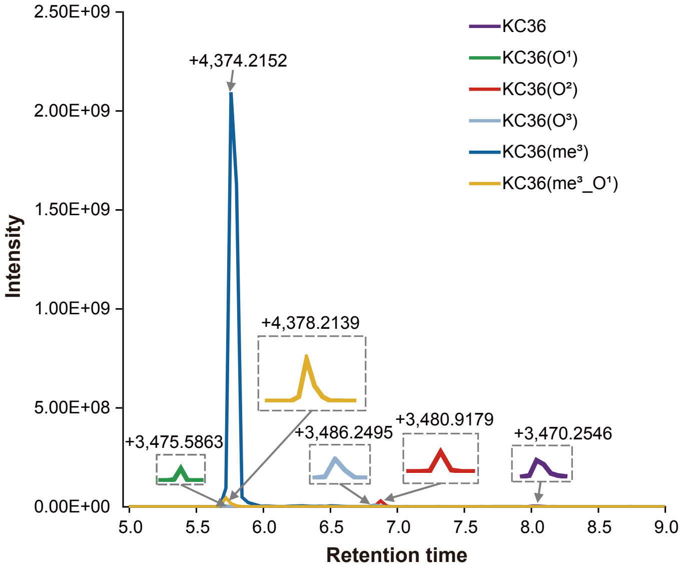


**Supplementary information, Fig. S2. SEC-MALS, XL-MS and LC-MS/MS analyses. a,** The molecular weight of apo Rpd3S was determined by SEC-MALS. The weight-average molar mass for the complex is plotted versus the elution volume, showing molar-mass values

over the peak width. The right table shows the molecular weights of Rpd3S subunits and the theoretical molar mass of Rpd3S containing two copies of Rco1 and Eaf3 is 513.90 kDa, which is similar to 527.7 kDa of the experimental molar mass. **b,** Circular plot displaying all the identified lysine-lysine inter-subunit crosslinks obtained by XL-MS for apo Rpd3S. **c,** LC-MS/MS extracted ion chromatograms of tryptic H3$K_C$36 and H3$K_C$36-me$^3$ peptides (-KSAPATGGVCKPHR-), peaks with different types of cysteine oxidation were also included. The charge state and m/z of each peptide were labelled above the peaks.
