# Supplementary material for: Structural basis of nucleosome deacetylation and DNA linker tightening by Rpd3S histone deacetylase complex
Source: Cell Res. 2023 Sep 4;33(10):790–801. doi: 10.1038/s41422-023-00869-1 (PMC10542350; doi:10.1038/s41422-023-00869-1)
Supplement: Supplementary file 3 — Supplementary information, Fig. S3 [file 41422_2023_869_MOESM3_ESM.pdf]

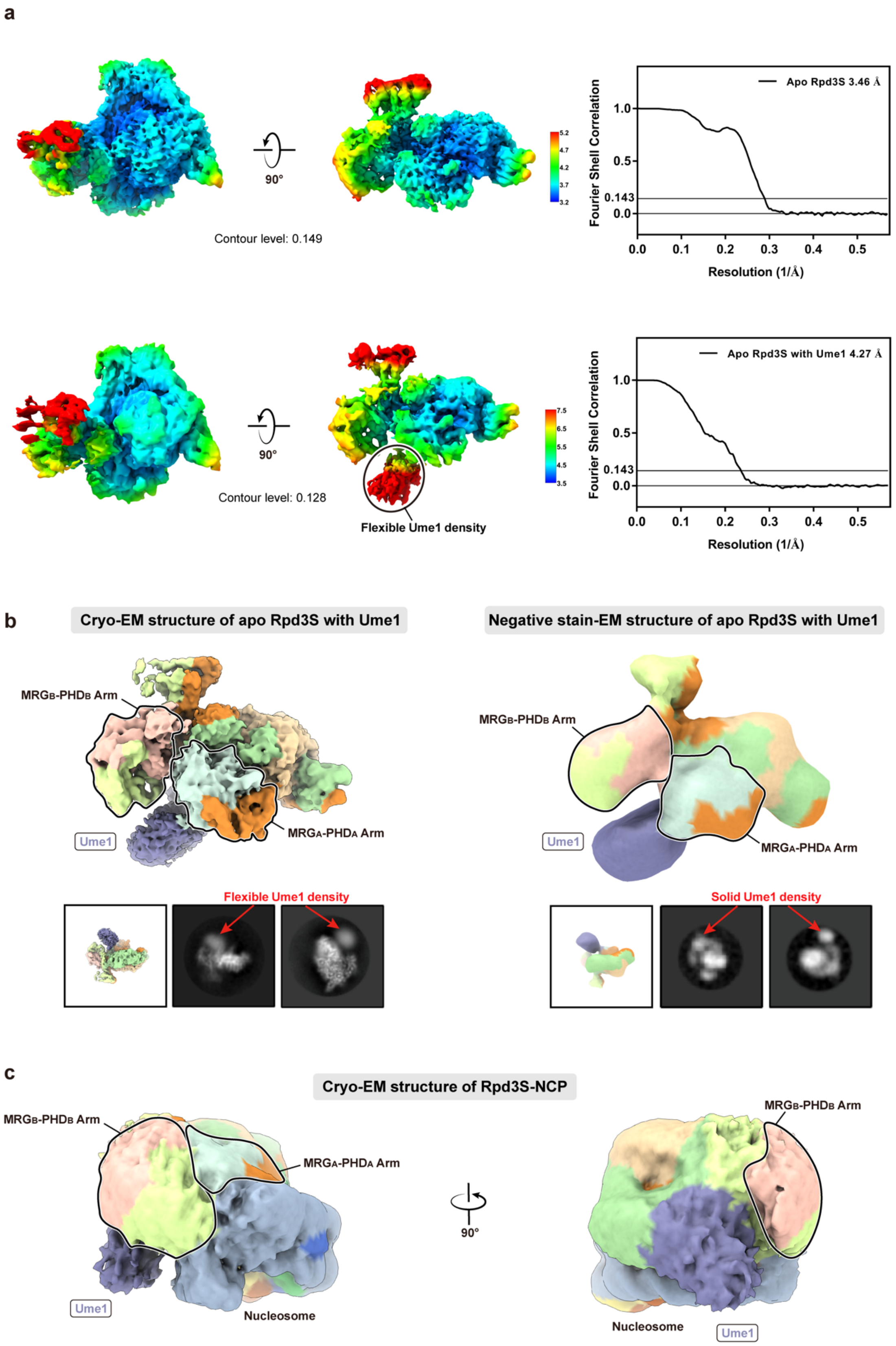

Supplementary information, Fig. S3. Cryo-EM processing and Ume1 position in Rpd3S.

a, Local resolution estimations and FSC curves of the cryo-EM maps of apo Rpd3S and apo

Rpd3S with Ume1 density. **b,** Cryo-EM and negative stain-EM 3D maps and 2D averages of apo Rpd3S with Ume1 density. The maps show Ume1 density located below MRG_B-PHD_B arm and close to MRG_A-PHD_A arm (upper panels). Ume1 density is more solid in 2D averages of negative stain-EM data (lower panel). **c,** Map of the Rpd3S-NCP complex showing Ume1 is underneath the MRG_B-PHD_B arm and close to the Sin3 base. Ume1 is colored purple.
