# Supplementary material for: Structural basis of nucleosome deacetylation and DNA linker tightening by Rpd3S histone deacetylase complex
Source: Cell Res. 2023 Sep 4;33(10):790–801. doi: 10.1038/s41422-023-00869-1 (PMC10542350; doi:10.1038/s41422-023-00869-1)
Supplement: Supplementary file 4 — Supplementary information, Fig. S4 [file 41422_2023_869_MOESM4_ESM.pdf]

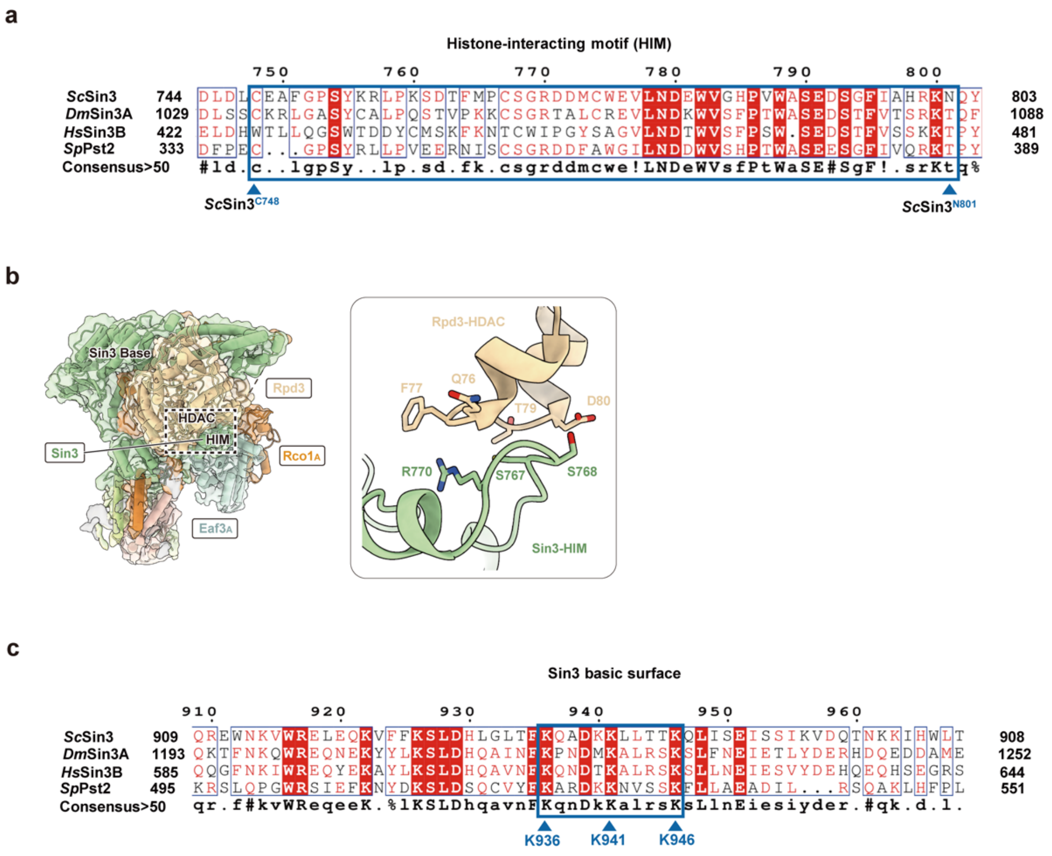

Supplementary information, Fig. S4. Sequence alignment and structural analysis of key Sin3 regions. a, Sequence alignment of equivalent Sin3 HIM regions in different species (*Sc, Dm, Hs* and *Sp*). **b,** Interactions between Rpd3 and HIM of Sin3. **c,** Sequence alignment of equivalent Sin3 basic surfaces in different species (*Sc, Dm, Hs* and *Sp*). The conserved residues are colored in red. *Saccharomyces cerevisiae, Drosophila melanogaster, Homo sapiens* and *Schizosaccharomyces pombe* are short for *Sc, Dm, Hs* and *Sp,* respectively.
