# Supplementary material for: Structural basis of nucleosome deacetylation and DNA linker tightening by Rpd3S histone deacetylase complex
Source: Cell Res. 2023 Sep 4;33(10):790–801. doi: 10.1038/s41422-023-00869-1 (PMC10542350; doi:10.1038/s41422-023-00869-1)
Supplement: Supplementary file 5 — Supplementary information, Fig. S5 [file 41422_2023_869_MOESM5_ESM.pdf]

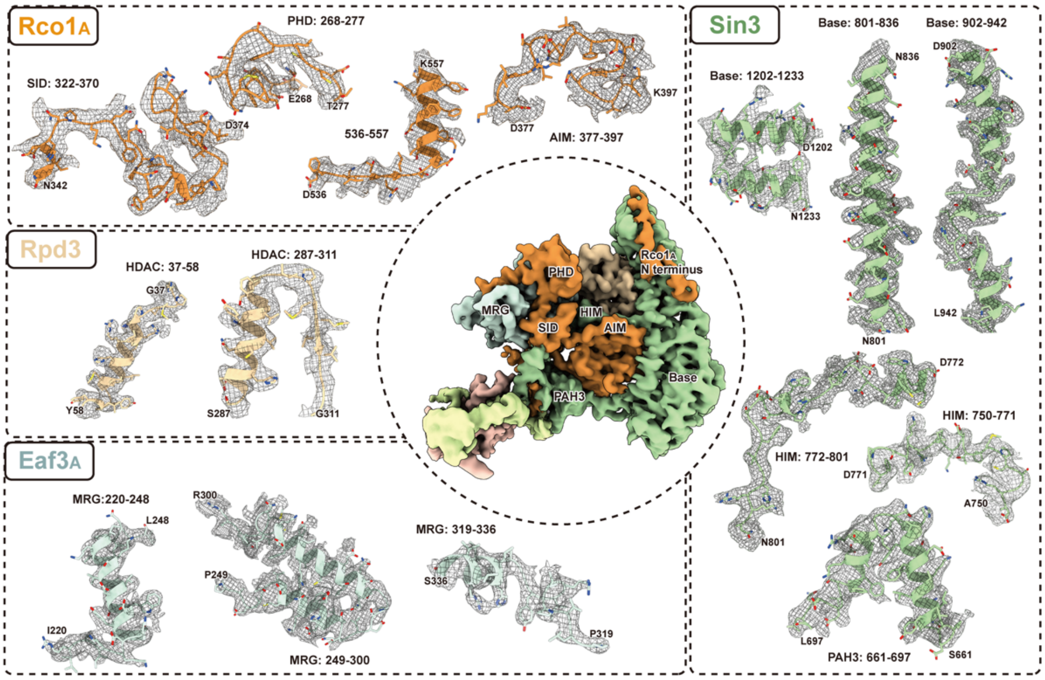

Supplementary information, Fig. S5. Local density maps of apo Rpd3S. Cryo-EM density maps and atomic models of selected key regions in apo Rpd3S. Key regions of Rpd3S subunits with cryo-EM densities are shown as meshes. The residues are shown as sticks.
