# Supplementary material for: Structural basis of nucleosome deacetylation and DNA linker tightening by Rpd3S histone deacetylase complex
Source: Cell Res. 2023 Sep 4;33(10):790–801. doi: 10.1038/s41422-023-00869-1 (PMC10542350; doi:10.1038/s41422-023-00869-1)
Supplement: Supplementary file 6 — Supplementary information, Fig. S6 [file 41422_2023_869_MOESM6_ESM.pdf]

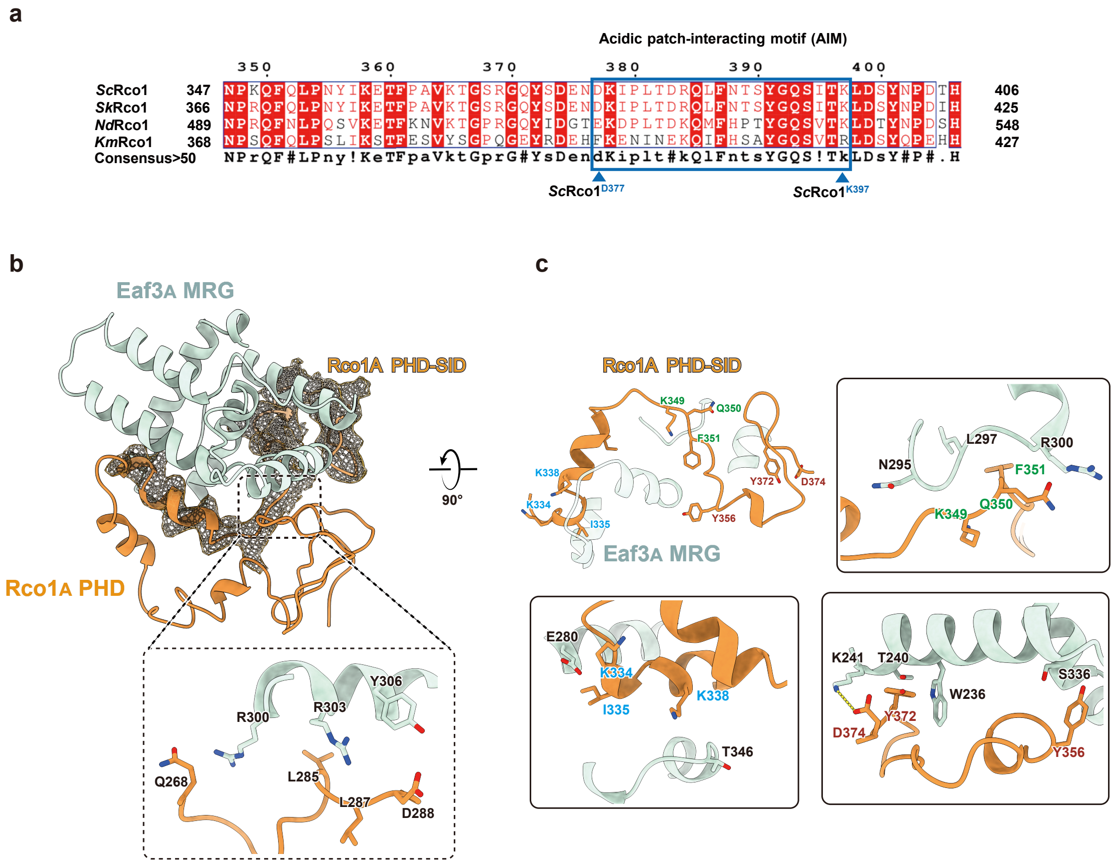

Supplementary information, Fig. S6. Sequence alignment and structure analysis of Rco1. **a,** Sequence alignment of equivalent Rco1 AIM in *Sc, Sk, Nd* and *Km*. The conserved residues are colored in red. *Saccharomyces cerevisiae, Saccharomyces kudriavzevii, Naumovozyma dairenensis* and *Kluyveromyces marxianus* are short for *Sc, Sk, Nd* and *Km,* respectively. **b,** Interactions between Eaf3$_A$ MRG and Rco1$_A$ PHD (left panel). The boxed region is enlarged for analysis. Rco1$_A$ PHD-SID with cryo-EM density was shown as mesh. **c,** Interactions between Eaf3$_A$ MRG and Rco1$_A$ PHD-SID (right panel). Key residues were colored green, blue and red in three enlarged interaction regions.
