# Supplementary material for: Structural basis of nucleosome deacetylation and DNA linker tightening by Rpd3S histone deacetylase complex
Source: Cell Res. 2023 Sep 4;33(10):790–801. doi: 10.1038/s41422-023-00869-1 (PMC10542350; doi:10.1038/s41422-023-00869-1)
Supplement: Supplementary file 7 — Supplementary information, Fig. S7 [file 41422_2023_869_MOESM7_ESM.pdf]

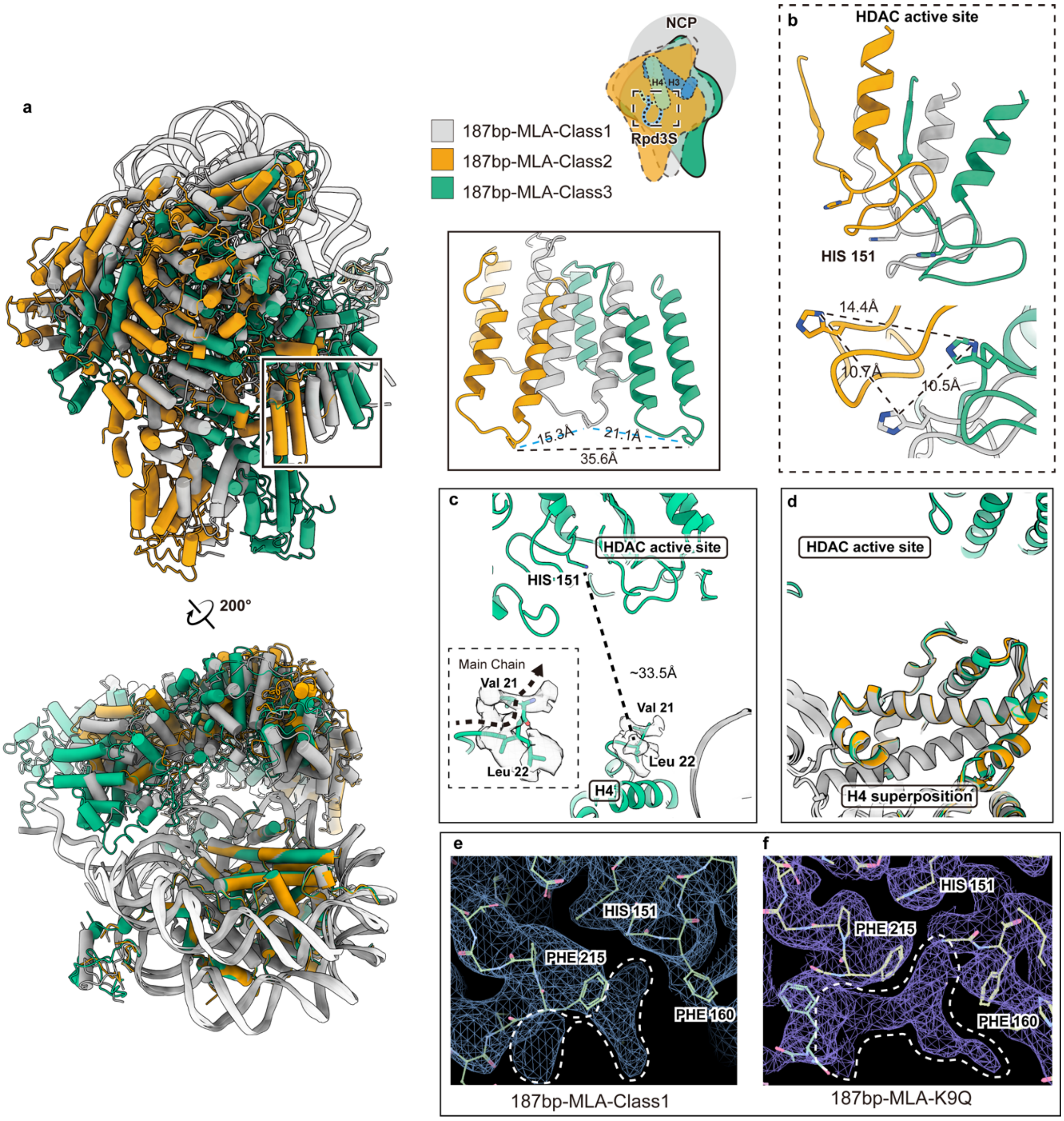

Supplementary information, Fig. S7. Rpd3S broadly engages the NCP at the SHL+2 position with slight local differential positioning. a, Rpd3S exhibits minor positional shifts of approximately 15-35 Å within various local regions. **b,** the active site of Rpd3 exhibits positional moves of approximately 10~15 Å. **c-d,** Rpd3S is able to target the H4 N-terminal tail. Specifically, the H4 N-terminal main chain of designated 187-MLA-class3 (corresponding density zoomed in) exhibits a distinctive trajectory leading toward the active site of Rpd3. **e-f,** Zoom-in view of the local density around the active site of Rpd3. Densities from a putatively unmodified histone tail (e) and H3K9Q tail (f) are outlined by dashed lines.
