# Supplementary material for: Structural basis of nucleosome deacetylation and DNA linker tightening by Rpd3S histone deacetylase complex
Source: Cell Res. 2023 Sep 4;33(10):790–801. doi: 10.1038/s41422-023-00869-1 (PMC10542350; doi:10.1038/s41422-023-00869-1)
Supplement: Supplementary file 8 — Supplementary information, Fig. S8 [file 41422_2023_869_MOESM8_ESM.pdf]

a

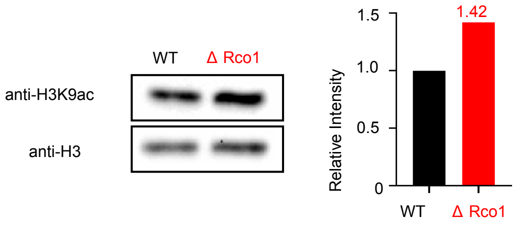

b

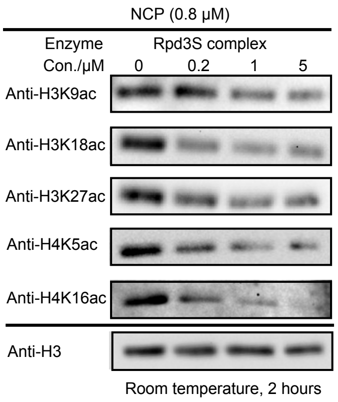

Supplementary information, Fig. S8. H3K9 is the deacetylation site of Rpd3S. a, The western blot shows increased global acetylation of H3K9 in the Rco1-deleted strain (BY4741 of budding yeast). **b,** Representative deacetylation assays of Rpd3S on nucleosomes. The histone octamer used in these assays was purified from a Rco1-deleted strain. Nucleosomes were reconstituted using this endogenous histone octamer with 187bp DNA. The reactions were performed by titration of Rpd3S (0, 0.2, 1, 5 μM) for 2 hours. The reaction products were identified using western blot.
