# Supplementary material for: Structural basis of nucleosome deacetylation and DNA linker tightening by Rpd3S histone deacetylase complex
Source: Cell Res. 2023 Sep 4;33(10):790–801. doi: 10.1038/s41422-023-00869-1 (PMC10542350; doi:10.1038/s41422-023-00869-1)
Supplement: Supplementary file 9 — Supplementary information, Fig. S9 [file 41422_2023_869_MOESM9_ESM.pdf]

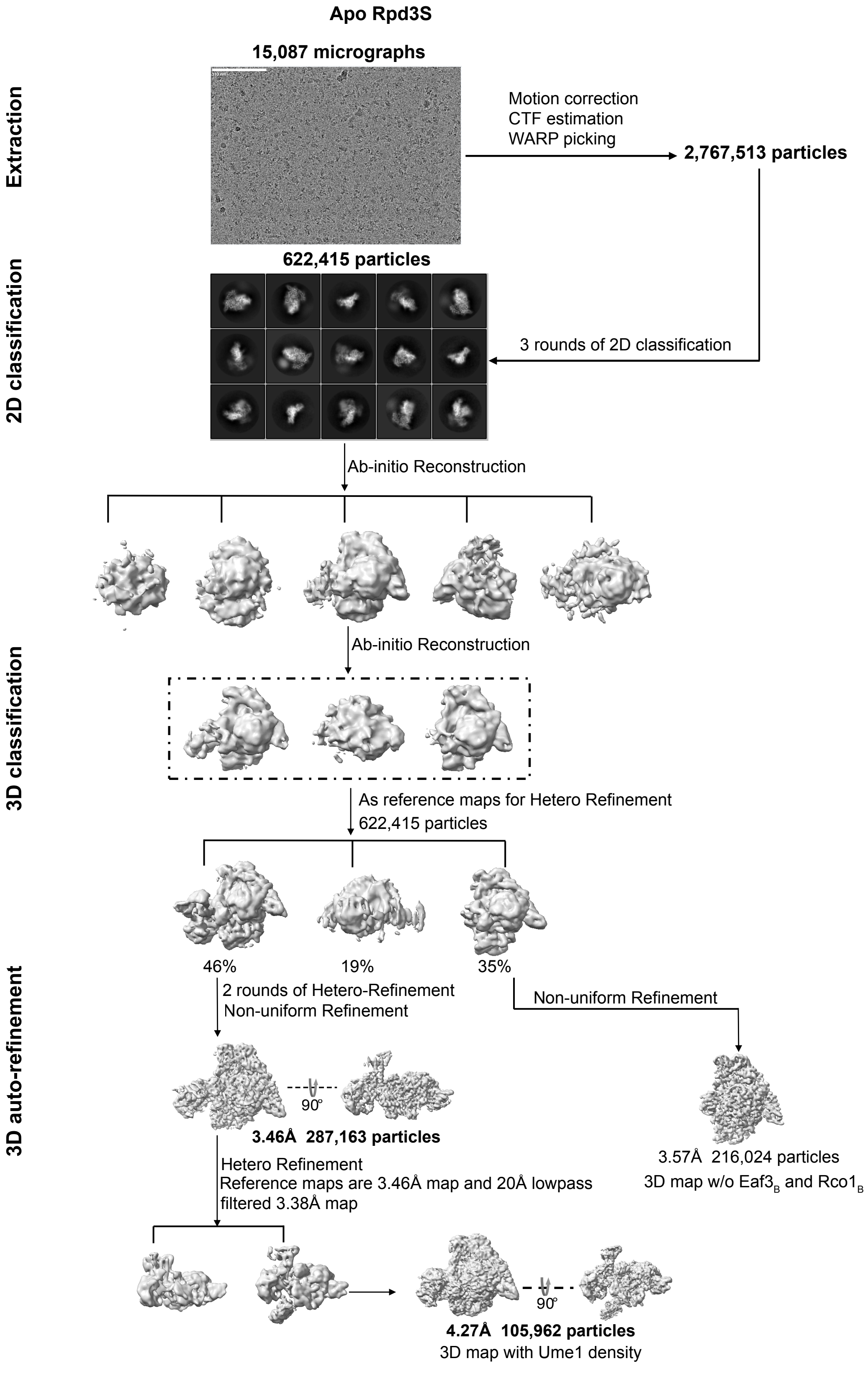

Supplementary information, Fig. S9. Data collection and image processing of apo Rpd3S.

Representative cryo-EM image, 2D classification and flow-charts of the cryo-EM images

processing and 3D reconstruction for apo Rpd3S in Relion and cryoSPARC. A lower-resolution 3D map is shown with Ume1 density. The overall resolution was estimated in Relion.
