# Supplementary material for: Structural basis of nucleosome deacetylation and DNA linker tightening by Rpd3S histone deacetylase complex
Source: Cell Res. 2023 Sep 4;33(10):790–801. doi: 10.1038/s41422-023-00869-1 (PMC10542350; doi:10.1038/s41422-023-00869-1)
Supplement: Supplementary file 10 — Supplementary information, Fig. S10 [file 41422_2023_869_MOESM10_ESM.pdf]

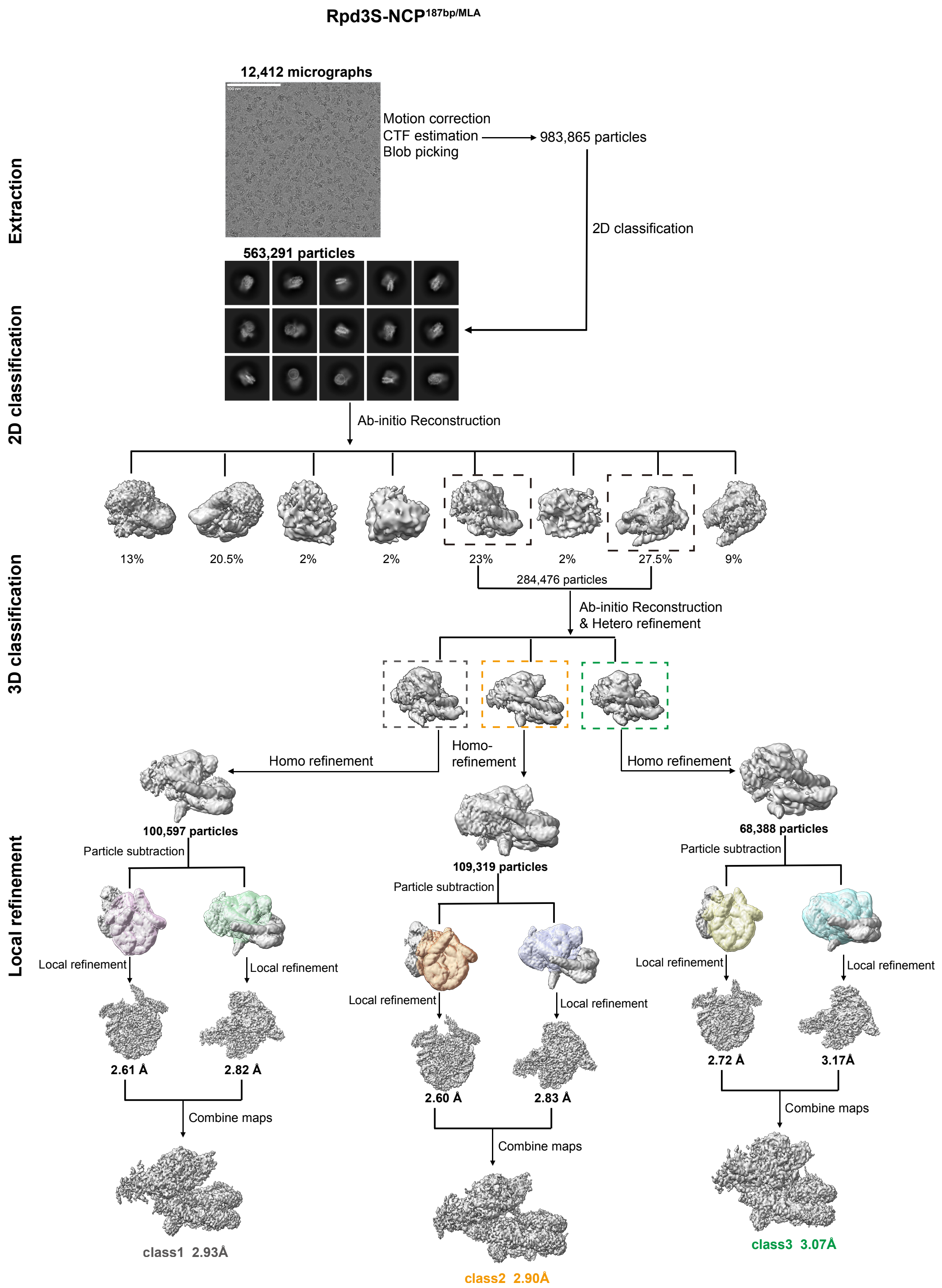

Supplementary information, Fig. S10. Data collection and image processing of Rpd3S-NCP$^{187bp/MLA}$. Representative cryo-EM images, 2D classifications and flow-charts of the cryo-EM images processing and 3D reconstruction for Rpd3S-NCP$^{187bp/MLA}$. Rpd3S and NCP were masked for particle subtraction and local refinement in cryoSPARC. Two focused maps were combined into one map in Chimera X.
