# Supplementary material for: Structural basis of nucleosome deacetylation and DNA linker tightening by Rpd3S histone deacetylase complex
Source: Cell Res. 2023 Sep 4;33(10):790–801. doi: 10.1038/s41422-023-00869-1 (PMC10542350; doi:10.1038/s41422-023-00869-1)
Supplement: Supplementary file 11 — Supplementary information, Fig. S11 [file 41422_2023_869_MOESM11_ESM.pdf]

Rpd3S-NCP$^{187bp/MLA/K9Q}$

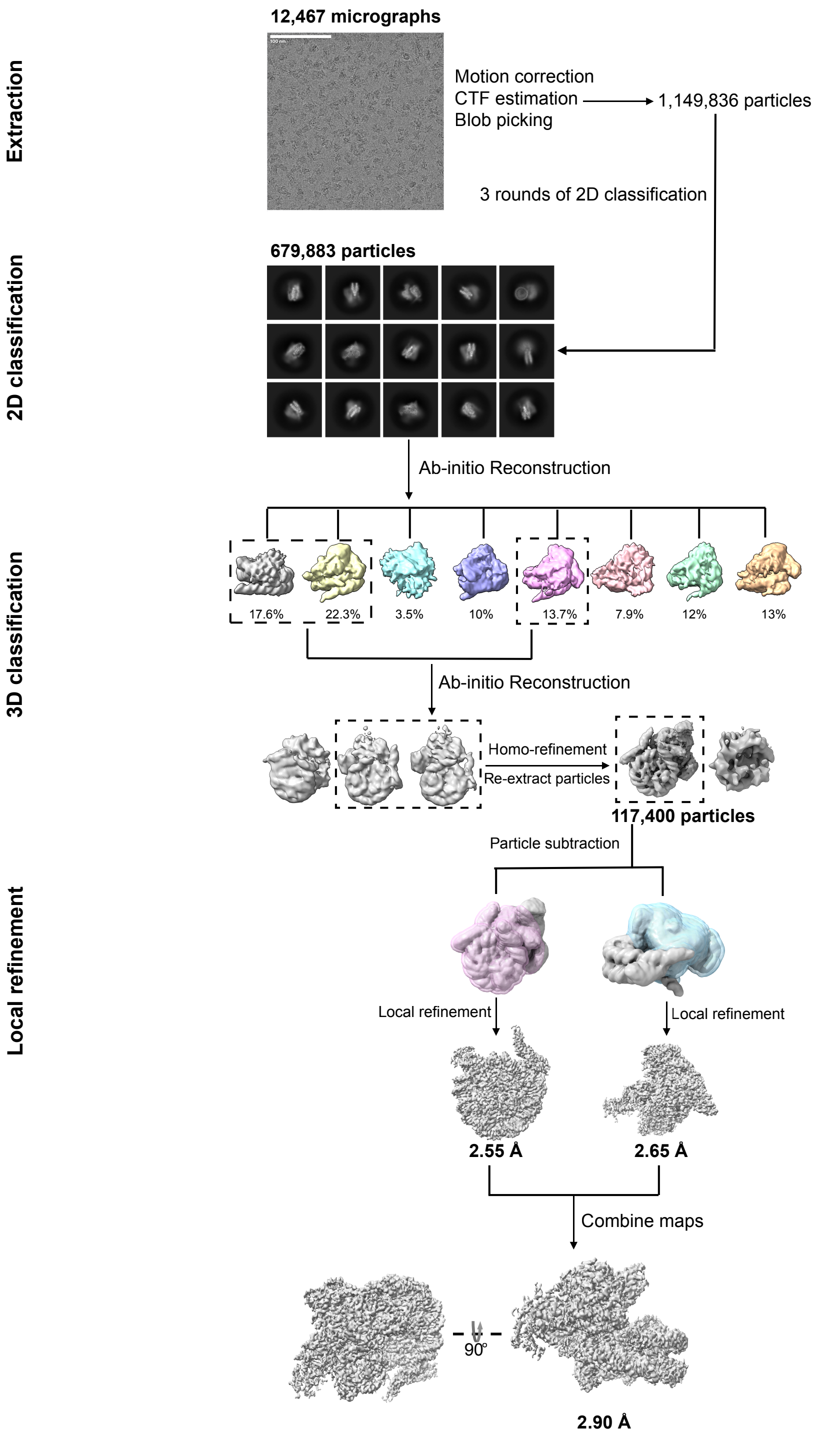

Supplementary information, Fig. S11. Data collection and image processing of Rpd3S-NCP$^{187bp/MLA/K9Q}$. Representative cryo-EM images, 2D classifications and flow-charts of the cryo-EM images processing and 3D reconstruction for Rpd3S-NCP$^{187bp/MLA/K9Q}$. Rpd3S and NCP were masked for particle subtraction and local refinement in cryoSPARC. Two focused

maps were combined into one map in Chimera X. The overall resolution was estimated in Relion.
