# Supplementary material for: Structural basis of nucleosome deacetylation and DNA linker tightening by Rpd3S histone deacetylase complex
Source: Cell Res. 2023 Sep 4;33(10):790–801. doi: 10.1038/s41422-023-00869-1 (PMC10542350; doi:10.1038/s41422-023-00869-1)
Supplement: Supplementary file 13 — Supplementary information, Fig. S13 [file 41422_2023_869_MOESM13_ESM.pdf]

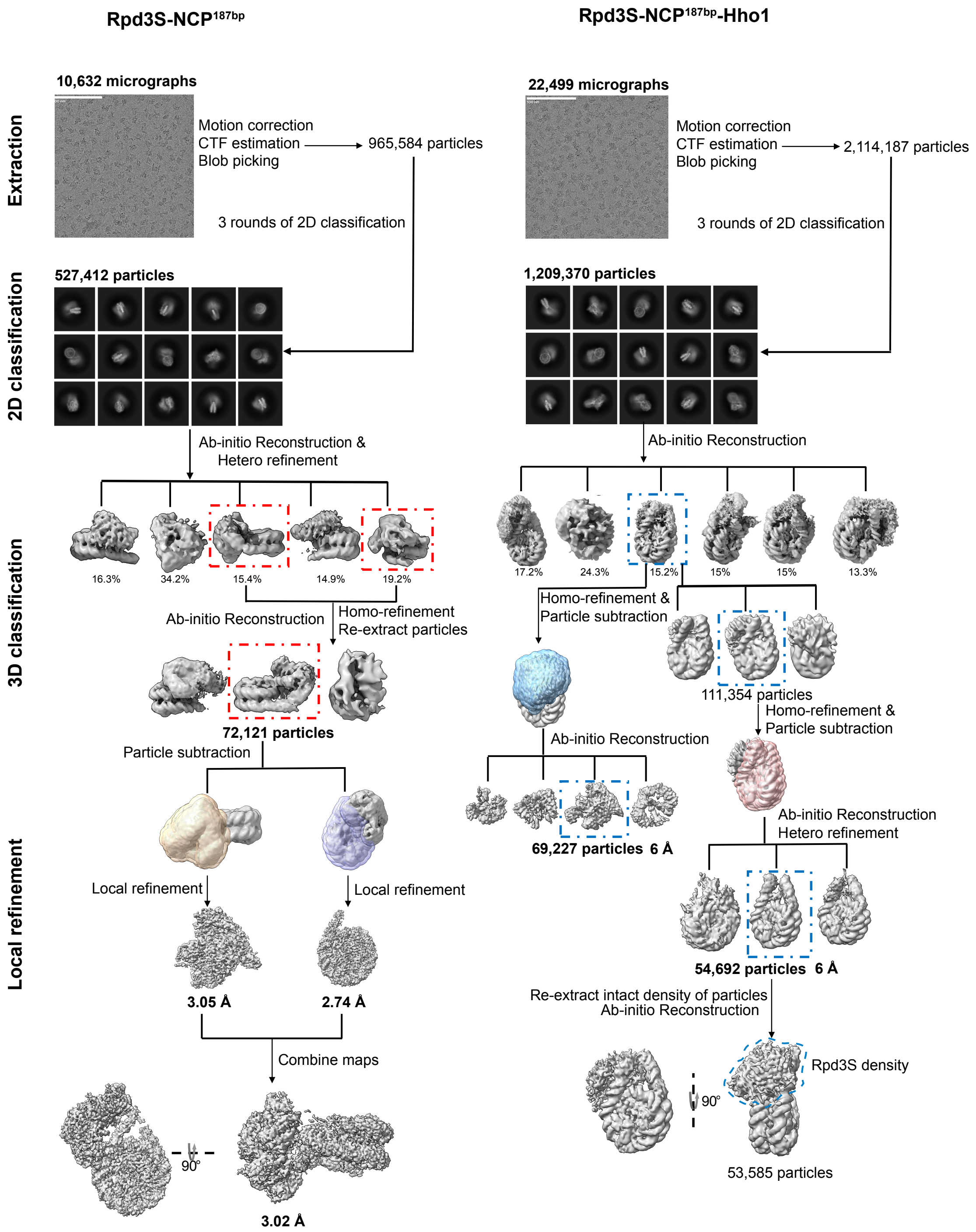

Supplementary information, Fig. S13. Data collection and image processing of Rpd3S-NCP^{187bp} and Rpd3S-NCP^{187bp}-Hho1. Representative cryo-EM images, 2D classifications and flow-charts of the cryo-EM images processing and 3D reconstruction for Rpd3S-NCP^{187bp} (left panel) and Rpd3S-NCP^{187bp}-Hho1 (right panel). In the workflow of Rpd3S-NCP^{187bp}, Rpd3S and NCP were masked for particle subtraction and local refinement in cryoSPARC. Two focused maps were combined into one map in Chimera X. The overall resolution of the combined maps was estimated in Relion. In the workflow of Rpd3S-NCP^{187bp}-Hho1, Rpd3S

and NCP-Hho1 were masked for particle subtraction and 3D reconstruction in cryoSPARC. NCP-Hho1 sub-particles were re-extracted with intact density particles followed by 3D reconstruction that resulted in a 3D map with Rpd3S density, indicating Rpd3S co-exists with Hho1 on NCP^{187bp}.
