# Supplementary material for: Structural basis of nucleosome deacetylation and DNA linker tightening by Rpd3S histone deacetylase complex
Source: Cell Res. 2023 Sep 4;33(10):790–801. doi: 10.1038/s41422-023-00869-1 (PMC10542350; doi:10.1038/s41422-023-00869-1)
Supplement: Supplementary file 14 — Supplementary information, Fig. S14 [file 41422_2023_869_MOESM14_ESM.pdf]

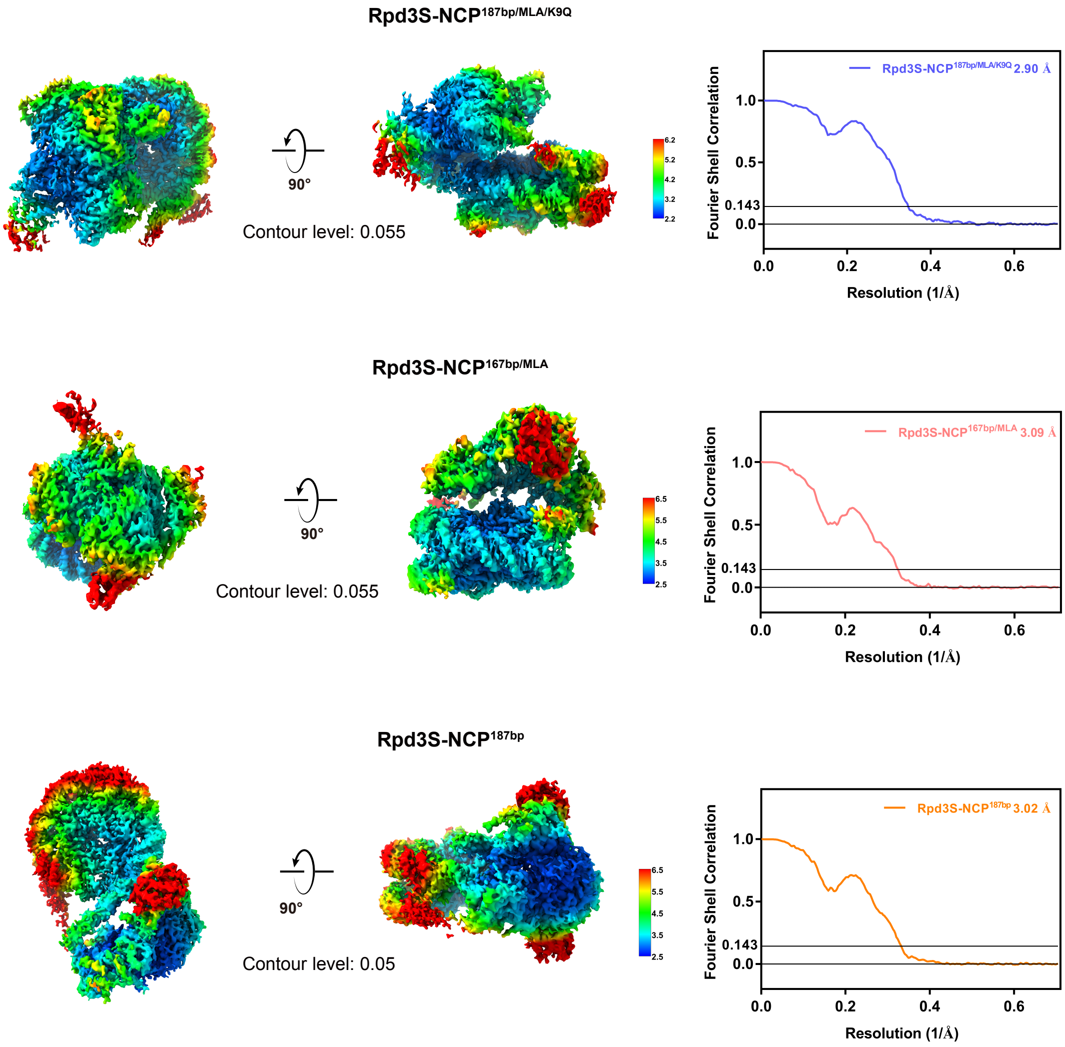

Supplementary information, Fig. S14. Local resolution estimations and FSC curves of Rpd3S-NCP$^{187bp/MLA/K9Q}$, Rpd3S-NCP$^{167bp/MLA}$ and Rpd3S-NCP^{187bp}. Local resolution estimations and Gold standard Fourier shell correlation (FSC) curves, showing the overall resolutions of 2.90 Å, 3.09 Å and 3.02 Å for the Rpd3S-NCP$^{187bp/MLA/K9Q}$, Rpd3S-NCP$^{167bp/MLA}$ and Rpd3S-NCP^{187bp} in Relion respectively. FSC curves with values of 0.143 are shown as the resolution criterion.
