# Supplementary material for: Structural basis of nucleosome deacetylation and DNA linker tightening by Rpd3S histone deacetylase complex
Source: Cell Res. 2023 Sep 4;33(10):790–801. doi: 10.1038/s41422-023-00869-1 (PMC10542350; doi:10.1038/s41422-023-00869-1)
Supplement: Supplementary file 15 — Supplementary information, Table S1 [file 41422_2023_869_MOESM15_ESM.pdf]

Supplementary information, Table. S1 | Cryo-EM data collection, refinement and validation statistics

		H3K9 deacetylation state	H3/H4 deacetylation states			Alternative deacetylation state	Linker tightening state	Rpd3S-Hho1 co-existing state
	Apo Rpd3S (EMD-37096,PDB 8KC7)	Rpd3S-NCP$^{187bp/MLA/K9Q}$ (EMD-37123,PDB 8KD3)	Rpd3S-NCP$^{187bp/MLA}$ Class1 (EMD-37124,PDB 8KD4)	Rpd3S-NCP$^{187bp/MLA}$ Class2 (EMD-37125,PDB 8KD5)	Rpd3S-NCP$^{187bp/MLA}$ Class3 (EMD-37126,PDB 8KD6)	Rpd3S-NCP$^{167bp/MLA}$ (EMD-37127,PDB 8KD7)	Rpd3S-NCP^{187bp} (EMD-37122,PDB 8KD2)	NCP^{187bp}-Hho1
**Data collection and processing**								
Magnification	45,000	165,000	165,000	165,000	165,000	165,000	165,000	165,000
Voltage(kV)	200	300	300	300	300	300	300	300
Electron exposure($e^-/Å^2$)	60	50	50	50	50	50	50	50
Energy filter slit width(eV)	—	10	10	10	10	10	10	10
Defocus range(μm)	-0.8 to -2.2	-0.8 to -2.2	-0.8 to -2.2	-0.8 to -2.2	-0.8 to -2.2	-0.8 to -2.2	-0.8 to -2.2	-0.8 to -2.2
Pixel size(Å)	0.88	0.71	0.71	0.71	0.71	0.71	0.71	0.71
Symmetry imposed	C1	C1	C1	C1	C1	C1	C1	C1
Movies(no.)	15,087	12,467	12,412	12,412	12,412	11,990	10,632	22,499
Initial particle images(no.)	2,767,513	1,149,836	983,865	983,865	983,865	1,061,981	965,584	2,114,187
Final particle images(no.)	287,163	117,400	100,597	109,319	68,388	56,949	72,121	54,692
Map resolution(Å) FSC threshold 0.143	3.46	2.90	2.93	2.90	3.07	3.09	3.02	6
**Refinement**								
Initial model used (PDB code)	2LKM, 1C3P, 2N2H and 6XAW	2LKM, 1C3P, 2N2H, 6XAW and 4LD9	2LKM, 1C3P, 2N2H, 6XAW and 4LD9	2LKM, 1C3P, 2N2H, 6XAW and 4LD9	2LKM, 1C3P, 2N2H, 6XAW and 4LD9	2LKM, 1C3P, 2N2H, 6XAW and 4LD9	2LKM, 1C3P, 2N2H, 6XAW and 4LD9	4LD9 and 7PFX
Map sharpening B factor($Å^2$)	-137.78	-31.94	-45.19	-41.23	-45.26	-43.48	-38.68	
Model composition								
Non-hydrogen atoms	14,330	27,587	27,055	26,888	26,971	24,922	24715	
Protein/Nucleotide	1,748	2,562/328	2,497/328	2,500/319	2,488/328	2,299/305	2164/348	
R.m.s deviations								
Bond lengths(Å)	0.003	0.004	0.004	0.003	0.004	0.004	0.004	
Bond angles(°)	0.706	0.761	0.833	0.747	0.870	0.884	0.717	
**Validation**								
MolProbity score	1.89	1.74	1.78	1.73	1.72	1.77	1.73	
Clashscore	8.63	7.10	7.96	7.82	7.46	7.80	7.93	
Poor rotamers(%)	0.93	0.09	0.59	0.54	0.77	0.25	0.57	
Ramachandran plot								
Favored(%)	93.43	95.01	95.05	95.70	95.47	95.10	95.69	
Allowed(%)	6.57	4.99	4.95	4.30	4.53	4.90	4.31	
Disallowed(%)	0.00	0.00	0.00	0.00	0.00	0.00	0.00	
Model to map CC (mask, box, peaks, volume)	0.78/0.8/ 0.71/0.78	0.66/0.74/ 0.61/0.66	0.74/0.77/ 0.65/0.74	0.74/0.76/ 0.64/0.74	0.71/0.76/ 0.61/0.71	0.67/0.75/ 0.61/0.67	0.74/0.78/ 0.66/0.73	
