# Supplementary material for: Structural basis of nucleosome deacetylation and DNA linker tightening by Rpd3S histone deacetylase complex
Source: Cell Res. 2023 Sep 4;33(10):790–801. doi: 10.1038/s41422-023-00869-1 (PMC10542350; doi:10.1038/s41422-023-00869-1)
Supplement: Supplementary file 16 — Supplementary figure legend [file 41422_2023_869_MOESM16_ESM.pdf]

Supplementary information, Fig. S1. Purification and cryo-EM structure of apo Rpd3S. **a,** Rpd3S was purified using gel filtration and the peak fractions were subjected to SDS-PAGE for Coomassie blue staining. **b,** Purified apo Rpd3S and four truncated complexes, $Rpd3S^{\Delta Ume1}$, $Rpd3S^{\Delta Eaf3}$, $Rpd3S^{\Delta PAH1/2}$ and $Rpd3S^{\Delta PAH1/2/3}$. **c,** Structure of apo Rpd3S. Two views of the high-resolution map and ribbon representations of apo Rpd3S. PHD2 domain (residues 416-470) is labelled. The three dimensions of Rpd3S are 148 Å x 113 Å x 88 Å.

Supplementary information, Fig. S2. SEC-MALS, XL-MS and LC-MS/MS analyses. a, The molecular weight of apo Rpd3S was determined by SEC-MALS. The weight-average molar mass for the complex is plotted versus the elution volume, showing molar-mass values over the peak width. The right table shows the molecular weights of Rpd3S subunits and the theoretical molar mass of Rpd3S containing two copies of Rco1 and Eaf3 is 513.90 kDa, which is similar to 527.7 kDa of the experimental molar mass. **b,** Circular plot displaying all the identified lysine-lysine inter-subunit crosslinks obtained by XL-MS for apo Rpd3S. **c,** LC-MS/MS extracted ion chromatograms of tryptic $H3K_C36$ and $H3K_C36\text{-}me^3$ peptides (-KSAPATGGVCKPHR-), peaks with different types of cysteine oxidation were also included. The charge state and m/z of each peptide were labelled above the peaks.

Supplementary information, Fig. S3. Cryo-EM processing and Ume1 position in Rpd3S. **a,** Local resolution estimations and FSC curves of the cryo-EM maps of apo Rpd3S and apo Rpd3S with Ume1 density. **b,** Cryo-EM and negative stain-EM 3D maps and 2D averages of apo Rpd3S with Ume1 density. The maps show Ume1 density located below MRG_B-PHD_B arm and close to MRG_A-PHD_A arm (upper panels). Ume1 density is more solid in 2D averages of negative stain-EM data (lower panel). **c,** Map of the Rpd3S-NCP complex showing Ume1 is underneath the MRG_B-PHD_B arm and close to the Sin3 base. Ume1 is colored purple.

Supplementary information, Fig. S4. Sequence alignment and structural analysis of key Sin3 regions. a, Sequence alignment of equivalent Sin3 HIM regions in different species (*Sc, Dm, Hs* and *Sp*). **b,** Interactions between Rpd3 and HIM of Sin3. **c,** Sequence alignment of equivalent Sin3 basic surfaces in different species (*Sc, Dm, Hs* and *Sp*). The conserved residues are colored in red. *Saccharomyces cerevisiae, Drosophila melanogaster, Homo sapiens* and *Schizosaccharomyces pombe* are short for *Sc, Dm, Hs* and *Sp,* respectively.

Supplementary information, Fig. S5. Local density maps of apo Rpd3S. Cryo-EM density maps and atomic models of selected key regions in apo Rpd3S. Key regions of Rpd3S subunits with cryo-EM densities are shown as meshes. The residues are shown as sticks.

Supplementary information, Fig. S6. Sequence alignment and structure analysis of Rco1. **a,** Sequence alignment of equivalent Rco1 AIM in *Sc, Sk, Nd* and *Km*. The conserved residues are colored in red. *Saccharomyces cerevisiae, Saccharomyces kudriavzevii, Naumovozyma dairenensis* and *Kluyveromyces marxianus* are short for *Sc, Sk, Nd* and *Km,* respectively. **b,** Interactions between $Eaf3_A$ MRG and $Rco1_A$ PHD (left panel). The boxed region is enlarged for analysis. $Rco1_A$ PHD-SID with cryo-EM density was shown as mesh. **c,** Interactions between $Eaf3_A$ MRG and $Rco1_A$ PHD-SID (right panel). Key residues were colored green, blue and red in three enlarged interaction regions.

Supplementary information, Fig. S7. Rpd3S broadly engages the NCP at the SHL+2 position with slight local differential positioning. a, Rpd3S exhibits minor positional shifts of approximately 15-35 Å within various local regions. **b,** the active site of Rpd3 exhibits positional moves of approximately 10~15 Å. **c-d,** Rpd3S is able to target the H4 N-terminal tail. Specifically, the H4 N-terminal main chain of designated 187-MLA-class3 (corresponding density zoomed in) exhibits a distinctive trajectory leading toward the active site of Rpd3. **e-f,** Zoom-in view of the local density around the active site of Rpd3. Densities from a putatively unmodified histone tail (e) and H3K9Q tail (f) are outlined by dashed lines.

Supplementary information, Fig. S8. H3K9 is the deacetylation site of Rpd3S. a, The western blot shows increased global acetylation of H3K9 in the Rco1-deleted strain (BY4741 of budding yeast). **b,** Representative deacetylation assays of Rpd3S on nucleosomes. The histone octamer used in these assays was purified from a Rco1-deleted strain. Nucleosomes were reconstituted using this endogenous histone octamer with 187bp DNA. The reactions were performed by titration of Rpd3S (0, 0.2, 1, 5 μM) for 2 hours. The reaction products were identified using western blot.

Supplementary information, Fig. S9. Data collection and image processing of apo Rpd3S. Representative cryo-EM image, 2D classification and flow-charts of the cryo-EM images processing and 3D reconstruction for apo Rpd3S in Relion and cryoSPARC. A lower-resolution 3D map is shown with Ume1 density. The overall resolution was estimated in Relion.

Supplementary information, Fig. S10. Data collection and image processing of Rpd3S-NCP$^{187bp/MLA}$. Representative cryo-EM images, 2D classifications and flow-charts of the cryo-EM images processing and 3D reconstruction for Rpd3S-NCP$^{187bp/MLA}$. Rpd3S and NCP were masked for particle subtraction and local refinement in cryoSPARC. Two focused maps were combined into one map in Chimera X.

Supplementary information, Fig. S11. Data collection and image processing of Rpd3S-NCP$^{187bp/MLA/K9Q}$. Representative cryo-EM images, 2D classifications and flow-charts of the cryo-EM images processing and 3D reconstruction for Rpd3S-NCP$^{187bp/MLA/K9Q}$. Rpd3S and NCP were masked for particle subtraction and local refinement in cryoSPARC. Two focused maps were combined into one map in Chimera X. The overall resolution was estimated in Relion.

Supplementary information, Fig. S12. Data collection and image processing of Rpd3S-NCP$^{167bp/MLA}$. Representative cryo-EM images, 2D classifications and flow-charts of the cryo-EM images processing and 3D reconstruction for Rpd3S-NCP$^{167bp/MLA}$. Rpd3S and NCP were masked for particle subtraction and local refinement in cryoSPARC. Two focused maps were combined into one map in Chimera X. The overall resolution of the combined maps was estimated in Relion.

Supplementary information, Fig. S13. Data collection and image processing of Rpd3S-NCP^{187bp} and Rpd3S-NCP^{187bp}-Hho1. Representative cryo-EM images, 2D classifications and flow-charts of the cryo-EM images processing and 3D reconstruction for Rpd3S-NCP^{187bp} (left panel) and Rpd3S-NCP^{187bp}-Hho1 (right panel). In the workflow of Rpd3S-NCP^{187bp}, Rpd3S and NCP were masked for particle subtraction and local refinement in cryoSPARC. Two focused maps were combined into one map in Chimera X. The overall resolution of the combined maps was estimated in Relion. In the workflow of Rpd3S-NCP^{187bp}-Hho1, Rpd3S and NCP-Hho1 were masked for particle subtraction and 3D reconstruction in cryoSPARC. NCP-Hho1 sub-particles were re-extracted with intact density particles followed by 3D reconstruction that resulted in a 3D map with Rpd3S density, indicating Rpd3S co-exists with Hho1 on NCP^{187bp}.

Supplementary information, Fig. S14. Local resolution estimations and FSC curves of Rpd3S-NCP$^{187bp/MLA/K9Q}$, Rpd3S-NCP$^{167bp/MLA}$ and Rpd3S-NCP^{187bp}. Local resolution

estimations and Gold standard Fourier shell correlation (FSC) curves, showing the overall resolutions of 2.90 Å, 3.09 Å and 3.02 Å for the Rpd3S-NCP$^{187bp/MLA/K9Q}$, Rpd3S-NCP$^{167bp/MLA}$ and Rpd3S-NCP^{187bp} in Relion respectively. FSC curves with values of 0.143 are shown as the resolution criterion.

Supplementary information, Table. S1 | Cryo-EM data collection, refinement and validation statistics.
